# Supplementary material for: Experiences of supported isolation in returning travellers during the early COVID-19 response: a qualitative interview study
Source: BMJ Open. 2021 Jul 23;11(7):e050405. doi: 10.1136/bmjopen-2021-050405 (PMC8313304; doi:10.1136/bmjopen-2021-050405)
Supplement: Supplementary data [file bmjopen-2021-050405supp002.pdf]

Appendix 2: Topic guide with questions relating to participants' experiences during and after supported isolation

During supported isolation

Where did you undergo supported isolation?

- Prompts:
    - If you underwent supported isolation at Arrowe Park, what date did you leave?

What were your thoughts about undergoing supported isolation?

- Prompts:
    - Did you understand why you were being put into supported isolation?
    - Did you have any concerns about undergoing supported isolation?
    - If so what were your concerns?
    - Did you think there were any benefits to you of undergoing supported isolation?

Tell me about your experience of undergoing supported isolation.

- Prompts:
    - What has it involved?
    - How has it been?

Were you aware of anyone who was in supported isolation with you having symptoms/ having the coronavirus?

- Prompts:
    - If so, what did you think/ how did you react?

In general, how do you feel the supported isolation process was managed at Arrowe Park/ Kents Hill Park?

Did you feel that staff/ authorities treated you fairly whilst you were in supported isolation?

- Prompts:
    - If so, why?
    - If not, why?

How did you feel about staff/ authority ability to successfully manage the supported isolation process?

Did you trust that the supported isolation process was being managed effectively?

Was there anything that could have been done to improve the way the supported isolation process was managed?

How well do you think staff/ authorities communicated with you whilst you were at Arrowe Park/ Kents Hill Park?

Did staff answer any questions you had?

Did you feel that you were provided with information that you needed?

- Prompts:
  - Were you provided with information about the incident?
  - Were you provided with information about what actions you should take?
  - Were you provided with information about why you were being asked to take certain actions?
  - Was there information you would have liked/ needed that you didn't receive? If so, what?

Did you get the opportunity to communicate with anyone outside of the supported isolation facility?

- Prompts:
  - If so, with who?
  - If so, how did you communicate with them (e.g. phone, social media, email etc)?
  - If so, how often?

Were you willing to undergo supported isolation?

- Prompts:
  - If so why?
  - If not why?
  - If not, what would have made it more likely you would comply?

How do you feel towards the other people who were in supported isolation with you?

- Prompts:
  - Did you feel any connection with them (e.g. bond, shared fate etc)
  - Were you concerned about the possibility of being infected by others?

Did you spend much time with them/ interact much with them?

Did your feelings about the other people affected change over the course of your time in supported isolation?

How did those in supported isolation behave towards each other?

Did you trust that other individuals in supported isolation would behave appropriately?

Did you see anyone refuse to undergo supported isolation?

- Prompts:
  - If so, what action did authorities take?
  - How did other people react?

Post-supported isolation

How has life been for you since leaving supported isolation?

Have you been given follow up information?

Overall, what do you think about the way the Government is managing this outbreak?

Do you feel you have experienced any unhelpful responses by others since undergoing supported isolation?

- Prompts:
  - Do you feel that people have reacted differently to you as a result of your having undergone supported isolation?
  - Do you feel that people have avoided you as a result of your having undergone supported isolation?

Is there anything else you'd like to say that hasn't been covered here?
