# Supplementary material for: Experiences of supported isolation in returning travellers during the early COVID-19 response: a qualitative interview study
Source: BMJ Open. 2021 Jul 23;11(7):e050405. doi: 10.1136/bmjopen-2021-050405 (PMC8313304; doi:10.1136/bmjopen-2021-050405)
Supplement: Author's manuscript [file bmjopen-2021-050405.draft_revisions.pdf]

BMJ Open is committed to open peer review. As part of this commitment we make the peer review history of every article we publish publicly available.

When an article is published we post the peer reviewers' comments and the authors' responses online. We also post the versions of the paper that were used during peer review. These are the versions that the peer review comments apply to.

The versions of the paper that follow are the versions that were submitted during the peer review process. They are not the versions of record or the final published versions. They should not be cited or distributed as the published version of this manuscript.

BMJ Open is an open access journal and the full, final, typeset and author-corrected version of record of the manuscript is available on our site with no access controls, subscription charges or pay-per-view fees (http://bmjopen.bmj.com).

If you have any questions on BMJ Open's open peer review process please email info.bmjopen@bmj.com

BMJ Open

Experiences of supported isolation in returning travellers during the early COVID-19 response: an interview study

Journal:	*BMJ Open*
Manuscript ID	bmjopen-2021-050405
Article Type:	Original research
Date Submitted by the Author:	22-Feb-2021
Complete List of Authors:	Carter, Holly; Public Health England Porton, Behavioural Science and Insights Unit<br>Weston, Dale; Public Health England Porton, Emergency Response Department<br>Greenberg , N; King's College London, King's Centre for Military Health Research, Department of Psychological Medicine<br>Oliver, Isabel; Public Health England, Field Epidemiology Services<br>Robin, Charlotte; Public Health England, Field Epidemiology<br>Rubin, GJ; King's College London,<br>Wessely, Simon; Institute of Psychiatry, King's Centre for Military Health Research<br>Amlot, Richard; Public Health England Porton, Emergency Response Department
Keywords:	COVID-19, PUBLIC HEALTH, QUALITATIVE RESEARCH

SCHOLARONE™
Manuscripts

I, the Submitting Author has the right to grant and does grant on behalf of all authors of the Work (as defined in the below author licence), an exclusive licence and/or a non-exclusive licence for contributions from authors who are: i) UK Crown employees; ii) where BMJ has agreed a CC-BY licence shall apply, and/or iii) in accordance with the terms applicable for US Federal Government officers or employees acting as part of their official duties; on a worldwide, perpetual, irrevocable, royalty-free basis to BMJ Publishing Group Ltd ("BMJ") its licensees and where the relevant Journal is co-owned by BMJ to the co-owners of the Journal, to publish the Work in this journal and any other BMJ products and to exploit all rights, as set out in our licence.

The Submitting Author accepts and understands that any supply made under these terms is made by BMJ to the Submitting Author unless you are acting as an employee on behalf of your employer or a postgraduate student of an affiliated institution which is paying any applicable article publishing charge ("APC") for Open Access articles. Where the Submitting Author wishes to make the Work available on an Open Access basis (and intends to pay the relevant APC), the terms of reuse of such Open Access shall be governed by a Creative Commons licence – details of these licences and which Creative Commons licence will apply to this Work are set out in our licence referred to above.

Other than as permitted in any relevant BMJ Author's Self Archiving Policies, I confirm this Work has not been accepted for publication elsewhere, is not being considered for publication elsewhere and does not duplicate material already published. I confirm all authors consent to publication of this Work and authorise the granting of this licence.

For peer review only

Experiences of supported isolation in returning travellers during the early COVID-19 response: an interview study

Authors: H Carter (0000-0002-2084-7263), D Weston, N Greenberg, I Oliver, C Robin, GJ Rubin, S Wessely, R Amlôt.

Behavioural Science and Insights Unit, Public Health England, Porton Down, Salisbury, SP4 0JG, UK

H Carter

Principal Behavioural Scientist

Behavioural Science and Insights Unit, Public Health England, Porton Down, Salisbury, SP4 0JG, UK

D Weston

Principal Behavioural Scientist

King's College London, Strand, London, WC2R 2LS, UK

N Greenberg

Professor of Defence Mental Health

National Infection Service, Public Health England, Bristol, UK

I Oliver

Director National Infection Service

Behavioural Science and Insights Unit, Public Health England, Porton Down, Salisbury, SP4 0JG, UK

C Robin

Behavioural Science Team Leader

Department of Psychological Medicine, King's College London, Weston Education Centre, Cutcombe Road, London, SE5 9RJ, UK

GJ Rubin

Reader in the Psychology of Emerging Health Risks

Department of Psychological Medicine, King's College London, Weston Education Centre, Cutcombe Road, London, SE5 9RJ, UK

S Wessely

Professor of Psychological Medicine

Behavioural Science and Insights Unit, Public Health England, Porton Down, Salisbury, SP4 0JG, UK

R Amlôt

Head of Behavioural Science

Correspondence to: H Carter holly.carter@phe.gov.uk, Behavioural Science and Insights Unit, Public Health England, Porton Down, Salisbury, SP4 0JG, UK.

For peer review only - http://bmjopen.bmj.com/site/about/guidelines.xhtml

Abstract

Objectives: To understand the experiences of those who underwent supported isolation as part of the response to the COVID-19 pandemic, after returning to the UK from Wuhan, China.

Design: We used semi-structured interviews to capture participants' experiences and perceptions of supported isolation.

Setting: Telephone interviews carried out within approximately one month of an individual leaving supported isolation.

Participants: 26 people who underwent supported isolation at either Arrowe Park Hospital (n = 18) or Kents Hill Park Conference Centre (n = 8) after being repatriated from Wuhan in January – February 2020.

Results: Participants were willing to undergo supported isolation because they understood that it would protect themselves and others. Positive treatment by staff was fundamental to participants' willingness to comply with isolation procedures. Despite the high level of compliance, participants expressed some uncertainty about what the process would involve.

Conclusions: As hotel quarantine is introduced across the UK for international arrivals, our findings suggest that those in charge should: communicate effectively before, during and after quarantine, emphasising why quarantine is important and how it will protect others; avoid enforcement and focus on supporting and promoting voluntary compliance; facilitate shared social experiences for those in quarantine; and ensure all necessary supplies are provided. Doing so will increase adherence and reduce any negative effects on wellbeing.

Strengths and limitations of this study

- Supported isolation, or quarantine, is a key public health intervention that can be used to control the spread of COVID-19.
- To our knowledge, the present study is the first research conducted with individuals during and immediately following their supported isolation in the UK as part of the COVID-19 response.
- We used semi-structured interviews to understand the experiences of a sample of people (n = 26) who underwent supported isolation, and to generate recommendations for supported isolation in similar contexts.
- Our findings are highly topical given the recent requirement for travellers to the UK to isolate within hotel accommodation.
- It was not possible to interview everyone who underwent supported isolation, and we were only able to interview those who had a good understanding of English.

For peer review only

Introduction

The first cases of a novel strain of coronavirus (SARS-CoV-2) were detected in Wuhan, China, in December 2019. On 31st January 2020, British Nationals living in Wuhan were offered repatriation to the UK. 93 returned on two chartered flights. In order to be repatriated all had to agree to undergo 14 days of 'supported isolation' (i.e. quarantine). This took place in an accommodation block at Arrowe Park Hospital in the Wirral [1]. A further 118 people returning from Wuhan underwent supported isolation at Kents Hill Park Conference Centre, Milton Keynes. All supported isolation ended by 23rd February 2020 [2].

Supported isolation for returning travellers had, to our knowledge, never been used before within the UK. It was anticipated that the experience could have considerable psychological consequences for the individuals concerned, including potential post-traumatic stress, anger and confusion; consequences that may be affected by a range of stressors including information provision, stigma, and fear of infection [3]. Furthermore, supported isolation represents a unique social context in which relative strangers are placed in close quarters within a novel context and asked to adhere to recommended behaviours for a prolonged period. During emergencies, such social contexts can affect individuals' social identity, which can have consequences for adherence and psychological resilience [4,5,6]. Outside of the emergency response context, the emergence of strong social connections among strangers in close physical proximity has been associated with positive well-being related outcomes [7].

From 15th February 2021 those travelling to the UK from some other countries will be required to isolate in hotels for 10 days [8]. Policy around this isolation is focused on identifying the best ways to maximise compliance, with an increasing emphasis on enforcement [9]. Furthermore, with the COVID-19 pandemic ongoing, it is possible that supported isolation will be required in other contexts, such as to assist those with difficulty isolating at home [10] and to reduce household transmission [11]. It is therefore important to understand more about the way in which people experience supported isolation, so that this process can be optimised to increase adherence and mitigate any negative effects on wellbeing. We carried out a rapid mixed-methods study in which we interviewed individuals who underwent supported isolation at Arrowe Park Hospital and Kents Hill Park conference centre. To our knowledge, this is the first research conducted with individuals during and immediately following their supported isolation in this country. These experiences are once again topical in light of the upcoming policy on required hotel isolation.

Method

Ethical approval

Ethical approval was obtained from the Public Health England Research Ethics Governance Group (approval no. NR0187).

Patient and public involvement

Given the extremely rapid and responsive nature of this research, it was not possible to involve patients or the public in the development of the study and associated materials. However, staff at the supported isolation facilities were involved from the outset in planning the study and facilitating participant recruitment. Additionally, findings from this study will be shared with participants on publication.

Design

This study used semi-structured interviews to capture participants' experiences and perceptions of supported isolation. Interviews took place over the telephone, within one-month post-supported isolation. The study was designed and carried out in-line with consolidated criteria for reporting qualitative research (COREQ) guidelines [12] (see Appendix 1).

For peer review only - http://bmjopen.bmj.com/site/about/guidelines.xhtml

Participants

Participants underwent supported isolation in either Arrowe Park (n = 18) or Kents Hill Park (n = 8) in January and February 2020. The day before leaving supported isolation, participants were provided with an information sheet about the study by a member of staff at the facility. This included an invitation to take part in a survey (findings reported elsewhere), as well as the opportunity to take part in an interview. On leaving supported isolation, 69 people provided a contact email address, and all were invited to take part in an interview. Of these, 26 people (38%) consented to take part in an interview, this sample therefore represents 12.3% of the entire population who underwent supported isolation. Half of the participants (n = 13) were male and half (n = 13) were female. Participants ranged in age from 22 to 78.

Materials

An interview schedule was developed to capture in-depth information about individuals' experiences and perceptions of supported isolation, including their: overall experience (e.g. "Tell me about your experience of undergoing supported isolation"); willingness to undergo supported isolation (e.g. "Were you willing to undergo supported isolation"); perceptions of the way the supported isolation process was managed (e.g. "In general, how do you feel the supported isolation process was managed?"); perceptions of others' behaviour during supported isolation (e.g. "How did those in supported isolation behave towards each other?"); experiences after leaving supported isolation (e.g. "How has life been for you since leaving supported isolation?").

Procedure

Each interview took place within one month of leaving the supported isolation facility and lasted for approximately an hour. Interviews were carried out by behavioural scientists based at Public Health England or King's College London, all of whom were qualified to at least MSc level and had received training in carrying out interviews. Researchers did not establish a relationship with participants prior to carrying out the interview nor were participants made aware of any personal characteristics of the interviewer, aside from their place of work and the broad aims of the research. Interviews were carried out by both male and female members of the research team. Only the researcher and the participant were present during the interview. Interviews were recorded and subsequently transcribed. After taking part in an interview, participants received a debriefing statement which provided further information about the study, as well as sources of support that participants could access if required. Participants were informed that they could request a copy of the results but did not provide feedback on the findings.

Analysis

A framework approach was used to analyse the data. This is a type of thematic analysis that is commonly used within research that has implications for policy and practice [13]. An a priori thematic framework was developed, but themes were also allowed to emerge from the data. This analysis generated 12 key themes: compliance; feelings about undergoing supported isolation; risk perceptions around catching COVID-19; protective behaviours during supported isolation; management of supported isolation; treatment by staff and authorities; communication from staff; communication with those outside of supported isolation facilities; relationship with others within supported isolation; thoughts about others' behaviour during supported isolation; areas for improvement; feelings on leaving supported isolation. Analysis was carried out by hand by the first author, and each passage was coded into one or more of the identified themes. After analysing the 26 transcripts no new themes emerged, thus data saturation had been reached [14].

Results

Results are presented by theme below; supporting quotes are presented in Appendix 2.

For peer review only - http://bmjopen.bmj.com/site/about/guidelines.xhtml

Compliance

Most participants were willing to undergo supported isolation. They understood why supported isolation was necessary and why they were being asked to undergo it. In the few instances where participants did not want to comply, non-compliance took the form of breaking the rules inside the supported isolation facility (e.g. trying to obtain more alcohol than was allowed), but not trying to leave the supported isolation facility.

Feelings about undergoing supported isolation

As well as discussing their willingness to comply with supported isolation measures, participants also discussed their feelings about undergoing supported isolation more broadly.

Most participants felt that the positives of supported isolation outweighed the negatives. Positive aspects were grouped broadly into three themes: a belief that supported isolation protects family and friends as well as UK society; a belief that supported isolation would protect themselves, by ensuring they were in a safe place if they developed symptoms and that they would not be blamed in the event of an outbreak in the UK; and faith in the effective management of the supported isolation process.

Where participants expressed concerns these centred around uncertainty about what the process would involve, sometimes attributing this to lack of information being provided. Others were concerned that they would be bored or would be at increased risk of catching COVID-19. A few felt angry or frustrated about the process, because they didn't think it was necessary or believed it was a waste of time and resources.

Risk perceptions around catching COVID-19

Participants' reported different perceived risks of catching COVID-19 whilst in isolation. Some felt at low risk because they could take protective behaviours; that anyone displaying symptoms could be quickly isolated; and that everyone in the supported isolation facility underwent regular testing.

However, others were very worried due to other people having symptoms, and the need to sometimes be in close proximity to others. In general, most participants stated that their risk perception reduced over time in the facility, as people continued to test negative, and did not have any symptoms. The majority of participants noted that they felt most worried at the start of supported isolation process.

Protective behaviours during supported isolation

The majority of participants reported that they performed protective behaviours. The most common included staying in their own room, observing effective hand hygiene measures, and wearing a face mask. While most participants reported that they took at least some protective measures, those who took fewer measures often reported that this was due to their perception that the risk of catching COVID-19 during supported isolation was low.

Management of supported isolation

Most participants reported that they felt the whole process was well-managed. Reasons for this included that the process was well-organised, and that staff and management were willing to adapt procedures following negative feedback about the process.

Where participants did express concerns these often centred on provision of food, for example not receiving meals, poor food options, food being served uncovered, and food not being warm enough. Another area of management that participants suggested could be improved was around internal communication within and between organisations. For the most part, participants who provided negative feedback about the management of the supported isolation process felt that changes were made to address their concerns, and that the management of the supported isolation process improved as time went on.

For peer review only - http://bmjopen.bmj.com/site/about/guidelines.xhtml

Treatment by supported isolation staff and authorities

Overall, participants were extremely positive in their feedback about the way in which staff treated them. The staff were friendly and helpful, went out of their way to keep people happy, and provided people with anything that they asked for. A few participants mentioned that staff did not try to avoid them or treat them as if they were ill. A small number of participants specifically noted that staff achieved a good balance between promoting good public health, without making the process too restrictive.

Communication from staff during supported isolation

Participants were also overwhelmingly positive about the way in which members of staff communicated with them. Almost all participants talked about the daily newsletter that they received from staff and felt that this was an effective way of providing information about protective actions, timings of any activities, and testing. Similarly, participants noted that staff were proactive in their communications, calling regularly to check on each individual, and scheduling regular update meetings. Participants also felt that staff answered all their questions (or tried to) and were open and transparent in providing information.

Some expressed dissatisfaction at the somewhat old-fashioned methods of communication, inability of staff to answer some questions, and information not being provided in multiple languages.

Communication with those outside of supported isolation facilities

Most participants found it easy to communicate with those outside supported isolation and did so regularly. Several participants expressed how important this was in helping them to get through the supported isolation process. Additionally, some were able to carry on working during supported isolation, and this helped them to pass the time. A few participants also highlighted the benefit of local community groups who posted pictures of uplifting things.

On the other hand, some participants did note difficulties in communicating with those outside of supported isolation, and these typically related to having limited access to internet or poor phone signal.

Relationship with others within supported isolation

Where people felt a connection with others this was often due to a sense of camaraderie or shared experience. Some participants described how people supported and encouraged each other during the supported isolation process, stating that this helped people to get through the experience. This connection was facilitated by the formation of chat groups, and some level of freedom to socialise with others.

Where people did not feel a connection with others this was because they either didn't get the opportunity to interact much with others, or actively avoided it (due to fears about catching COVID-19).

Thoughts about others' behaviour during supported isolation

Most participants felt that they could trust others to behave appropriately and instances of uncooperative behaviour were rare or non-existent. A handful of participants noted isolated instances of uncooperative behaviour, but almost all said that the majority of people were friendly and cooperative.

Areas for improvement in supported isolation procedures

Some felt they would have liked more information about what supported isolation would involve. Others suggested it would have been beneficial to have more access to outside space and exercise facilities. Another common area for improvement was the food provided, with people suggesting that food options and quality could have been better.

For peer review only - http://bmjopen.bmj.com/site/about/guidelines.xhtml

Feelings after leaving supported isolation

Many participants felt happy and relieved to leave supported isolation and get back to normal. However, several participants stated that they struggled after leaving supported isolation. Some felt anxious or overwhelmed, with reasons including not being used to going outside, or being concerned about mixing with large numbers of people again. Others simply stated that they had generally struggled on leaving, or that they had experienced negative reactions from others.

The majority of participants did not receive follow up information, though a few did receive information about sources of further support. While some stated that they would not have expected to receive any additional information, others felt that this would have been helpful.

Discussion

This paper represents the first in-depth analysis of the experiences of those who underwent supported isolation in the UK during the first wave of the COVID-19 pandemic. Given that supported isolation is once again required in the management of COVID-19 [8,10,11], our findings should help facilitate optimised management.

Despite some initial concerns, including confusion about what the process would involve and fears of infection, all willingly complied with the voluntary supported isolation process. People understood why it was necessary and believed that doing so would protect themselves, their friends and family, and others in the UK; motivation for adherence was largely altruistic. Participants were overwhelmingly positive about their treatment by staff, communication from staff, and overall management of the supported isolation process. This was fundamental to participants' willingness to comply with the restrictions of their liberty. Our findings are in line with systematic reviews carried out at the start of the pandemic [3,15], as well as research into the management of other types of emergencies [4,16]. Crucially, participants believed their treatment by staff was legitimate, and they therefore chose to comply with supported isolation procedures; it is likely that compliance would have been much lower had staff attempted to enforce compliance [16].

There were mixed views as to whether people in isolation experienced a connection with each other. However, almost all reported that others were helpful and friendly. Additionally, a number of people developed a shared identity with others; for example, they talked about everyone being in it together or going through the same experience. Those that did develop a shared identity often reported that this helped them to get through the process. This is as would be expected based on previous research which suggests that when people experience a sense of shared identity with others, this promotes adherence to protective measures, resilience and well-being [4,5,7]. While a sense of shared social identity arose spontaneously in some instances, participants emphasised that being able to communicate with others (for example, via chat groups) enhanced the social support that they experienced. Promoting virtual interaction between those undergoing supported isolation may be beneficial for strengthening shared identity, facilitating provision of social support, and promoting resilience and well-being. Participants also highlighted how important it was that they were able to easily keep in touch with friends and family during the supported isolation process. Of particular interest was our finding that some participants reported negative experiences on leaving supported isolation. It may therefore be beneficial to prepare participants for possible psychosocial reactions prior to them leaving supported isolation and signpost them to sources of support.

Limitations

Approximately a third of participants who were contacted about this study agreed to take part. We have no information on those who did not participate, and it is possible that they differed on key variables. Of those that did participate we reached thematic saturation within the sample. Furthermore, participants were

For peer review only - http://bmjopen.bmj.com/site/about/guidelines.xhtml

aware of what was going on around theme, so the reports of very high compliance with supported isolation and other protective behaviours can be generalised to all those who were in quarantine. The same goes for the general finding that most people were friendly and cooperative. A second limitation is that only those who had a good understanding of English were interviewed. It is therefore possible that the experience differed for those who were less able to understand English; indeed, this was alluded to in some comments made by participants. A final limitation is that this study was jointly run by King's College London and Public Health England, and Public Health England also assisted with the management of the supported isolation process. Although the team carrying out this research were not associated with the management of the supported isolation process, it is possible that participants were aware that PHE played a role in managing the supported isolation process, and that this affected their responses during the interview.

Conclusion and Recommendations

The findings presented here, particularly when situated within the wider literature, generate several key recommendations that are particularly relevant given the upcoming requirement for travellers to isolate in hotels. The supported isolation carried out in January – February 2020 was designed to support those who were returning to the UK, and every effort was made to ensure that their experience was as positive as possible; as participants noted, staff could not do enough for them. Isolation in hotels is likely to be fundamentally different, with limited support from staff and an emphasis increasingly on enforcement rather than encouragement [9]. The reasons why people are travelling in the middle of a pandemic will also be different. The UK may find itself placing people into isolation who are more likely to experience distress such as those who are arriving to attend a funeral, are travelling due to a family crisis, or who do not speak English. We must also not forget that, unlike travellers placed into facilities at Arrowe Park or Kents Hill, returning travellers will now be asked to pay £1,500 each towards their isolation. It is therefore critical that those responsible for implementing policies on isolation requirements take into account the recommendations presented here; failure to do so is likely to reduce adherence to isolation and risks serious long-term impact on those involved.

Specific recommendations are: 1) prior to supported isolation, communicate with those affected about why isolation is necessary, how it will help to protect others, and what the process will involve. Given that compliance is often motivated by altruism, emphasising how isolation will protect others is crucial. Such communication will also reduce concerns related to uncertainty about the isolation process; 2) communicate effectively with those undergoing isolation, throughout the process. Communication should be open and honest, and information should include protective actions people should take, why taking such actions is effective, and how taking such actions protects oneself and others; 3) enforcement with isolation should be avoided wherever possible. Given the large numbers of people who may be required to isolate at one time it will not be possible to enforce adherence; attempting to do so is likely to be perceived as illegitimate, thereby reducing adherence and risking serious long term consequences for those involved; 4) facilitate and encourage development of shared identity among those undergoing supported isolation, via the formation of chat groups or other means of communication, that include staff managing the facilities. This type of shared social identity should encourage both adherence to supported isolation measures, and improved resilience during the supported isolation process; 5) ensure that all essential supplies (such as food, exercise facilities, ability to communicate with those outside isolation) are provided and are suitable for the needs of the traveller; 6) provide information prior to leaving supported isolation to help people to prepare to return to their normal lives. This should include information about emotions that people might experience, and sources of further support that people can access if required. It may also be beneficial to include in this information any ongoing expectations around adherence to protective behaviours.

For peer review only - http://bmjopen.bmj.com/site/about/guidelines.xhtml

Competing interest statement

All authors have completed the ICMJE uniform disclosure form and declare: HC, DW, IO, CR, and RA are current employees of Public Health England; GJR participates in the UK's Scientific Advisory Group for Emergencies and its subgroups.

Transparency declaration

The lead author affirms that this manuscript is an honest, accurate, and transparent account of the study being reported; that no important aspects of the study have been omitted; and that any discrepancies from the study as originally planned (and, if relevant, registered) have been explained.

Contributor statement

RA, HC, DW, NG, GJR, IO and SW conceived the study. HC, CR, DW and RA collected the data. HC carried out the analysis and wrote the first draft of the manuscript. All authors contributed to the design and implementation of the study, and to the writing of the manuscript.

Data availability statement

Data are available on request from holly.carter@phe.gov.uk.

Funding statement

This study was funded by the National Institute for Health Research Health Protection Research Unit (NIHR HPRU) in Emergency Preparedness and Response (grant number 200890), a partnership between Public Health England, King's College London and the University of East Anglia. DW, IO, CR and RA are supported by the NIHR HPRU in Behavioural Science and Evaluation, a partnership between Public Health England and the University of Bristol. CR is also supported by the NIHR HPRU in Emerging and Zoonotic Infections and NIHR HPRU in Gastrointestinal Infections. The views expressed are those of the authors and not necessarily those of the NIHR, Public Health England or the Department of Health and Social Care.

For peer review only

For peer review only - http://bmjopen.bmj.com/site/about/guidelines.xhtml

References

1. BBC News. *Coronavirus: Britons evacuated from Wuhan arrive at quarantine facility*. BBC News [newspaper on the Internet] 2020 Jan 31 [cited 2021 Feb 2]. Available from: https://www.bbc.co.uk/news/uk-51318691.

2. BBC News. *Coronavirus: Wuhan Britons end quarantine as cruise passengers isolate*. BBC News [newspaper on the Internet] 2020 Feb 23 [cited 2021 Feb 2]. Available from: https://www.bbc.co.uk/news/uk-51602986.

3. Brooks SK, Webster R, Smith LE, et al. The psychological impact of quarantine and how to reduce it: Rapid review of the evidence. *The Lancet* 2020;395(10227):912–920.

4. Carter H, Drury J, Rubin GJ, Williams R, Amlôt R. Applying crowd psychology to develop recommendations for the management of mass decontamination. *Health Security* 2015;13(1):45–53.

5. Drury J, Carter H, Cocking C, Ntontis E, Tekin Guven S, Amlôt R. Facilitating collective psychosocial resilience in the public in emergencies: twelve recommendations based on the social identity approach. *Frontiers in Public Health* 2019;7(141):Doi: 10.3389/fpubh.2019.00141.

6. Drury J, Carter H, Ntontis E, Tekin Guven S. Public behaviour in response to the COVID-19 pandemic: understanding the role of group processes. *BJPsych Open* 2020;1:1–6.

7. Howell JL, Koudenberg N, Loschelder DD, et al. Happy but unhealthy: The relationship between social ties and health in an emerging network. *European Journal of Social Psychology* 2014;44(6):612–621.

8. BBC News. *Covid: Quarantine hotel plans set to be announced*. BBC News [newspaper on the Internet] 2020 Jan 26 [cited 2021 Feb 2]. Available from: https://www.bbc.co.uk/news/uk-politics-55805575.

9. BBC News. *Covid-19: Travellers face £1,750 cost for England quarantine hotels*. BBC News [newspaper on the Internet] 2021 Feb 9 [cited 2021 Feb 9]. Available from: https://www.bbc.co.uk/news/uk-55995645 9th February 2021.

10. Gurdasani D, Bear L, Bogaert D, et al. The UK needs a sustainable strategy for COVID-19. *The Lancet* 2020;396:1800–1801.

11. Denford S, Morton K, Horwood J, de Garang R, Yardley L. Preventing within household transmission of COVID-19: Is the provision of accommodation feasible and acceptable? *medRxiv* 2020.

12. Tong A, Sainsbury P, Craig J. Consolidated criteria for reporting qualitative research (COREQ): a 32-item checklist for interviews and focus groups. *International Journal for Quality in Health Care* 2007;19(6):349-357.

13. Pope C, Ziebland S, Mays N. Analysing qualitative data. *BMJ* 2000;320:114–116.

14. Glaser BG, Strauss AL. *The discovery of grounded theory: strategies for qualitative theory*. New Brunswick: Aldine Transaction; 1967.

15. Webster RK, Brooks SK, Smith L, Woodland L, Wessely S, Rubin GJ. How to improve adherence with quarantine: Rapid review of the evidence. *Public Health* 2020;182:163–169.

For peer review only - http://bmjopen.bmj.com/site/about/guidelines.xhtml

16. Carter H, Drury J, Amlôt R. Social identity and intergroup relationships in the management of crowds during mass emergencies and disasters: recommendations for emergency planners and responders. *Policing: A Journal of Policy and Practice* 2018:1–14.

For peer review only

For peer review only - http://bmjopen.bmj.com/site/about/guidelines.xhtml

COREQ (COnsolidated criteria for REporting Qualitative research) Checklist

A checklist of items that should be included in reports of qualitative research. You must report the page number in your manuscript where you consider each of the items listed in this checklist. If you have not included this information, either revise your manuscript accordingly before submitting or note N/A.

Topic	Item No.	Guide Questions/Description	Reported on Page No.
**Domain 1: Research team and reflexivity**			
Personal characteristics			
Interviewer/facilitator	1	Which author/s conducted the interview or focus group?	
Credentials	2	What were the researcher's credentials? E.g. PhD, MD	
Occupation	3	What was their occupation at the time of the study?	
Gender	4	Was the researcher male or female?	
Experience and training	5	What experience or training did the researcher have?	
Relationship with participants			
Relationship established	6	Was a relationship established prior to study commencement?	
Participant knowledge of the interviewer	7	What did the participants know about the researcher? e.g. personal goals, reasons for doing the research	
Interviewer characteristics	8	What characteristics were reported about the inter viewer/facilitator? e.g. Bias, assumptions, reasons and interests in the research topic	
**Domain 2: Study design**			
Theoretical framework			
Methodological orientation and Theory	9	What methodological orientation was stated to underpin the study? e.g. grounded theory, discourse analysis, ethnography, phenomenology, content analysis	
Participant selection			
Sampling	10	How were participants selected? e.g. purposive, convenience, consecutive, snowball	
Method of approach	11	How were participants approached? e.g. face-to-face, telephone, mail, email	
Sample size	12	How many participants were in the study?	
Non-participation	13	How many people refused to participate or dropped out? Reasons?	
Setting			
Setting of data collection	14	Where was the data collected? e.g. home, clinic, workplace	
Presence of non-participants	15	Was anyone else present besides the participants and researchers?	
Description of sample	16	What are the important characteristics of the sample? e.g. demographic data, date	
Data collection			
Interview guide	17	Were questions, prompts, guides provided by the authors? Was it pilot tested?	
Repeat interviews	18	Were repeat inter views carried out? If yes, how many?	
Audio/visual recording	19	Did the research use audio or visual recording to collect the data?	
Field notes	20	Were field notes made during and/or after the inter view or focus group?	
Duration	21	What was the duration of the inter views or focus group?	
Data saturation	22	Was data saturation discussed?	
Transcripts returned	23	Were transcripts returned to participants for comment and/or	

For peer review only

Topic	Item No.	Guide Questions/Description	Reported on Page No.
		correction?	
**Domain 3: analysis and findings**			
Data analysis			
Number of data coders	24	How many data coders coded the data?	
Description of the coding tree	25	Did authors provide a description of the coding tree?	
Derivation of themes	26	Were themes identified in advance or derived from the data?	
Software	27	What software, if applicable, was used to manage the data?	
Participant checking	28	Did participants provide feedback on the findings?	
Reporting			
Quotations presented	29	Were participant quotations presented to illustrate the themes/findings? Was each quotation identified? e.g. participant number	
Data and findings consistent	30	Was there consistency between the data presented and the findings?	
Clarity of major themes	31	Were major themes clearly presented in the findings?	
Clarity of minor themes	32	Is there a description of diverse cases or discussion of minor themes?	

Developed from: Tong A, Sainsbury P, Craig J. Consolidated criteria for reporting qualitative research (COREQ): a 32-item checklist for interviews and focus groups. *International Journal for Quality in Health Care*. 2007. Volume 19, Number 6: pp. 349 – 357

Once you have completed this checklist, please save a copy and upload it as part of your submission. DO NOT include this checklist as part of the main manuscript document. It must be uploaded as a separate file.

For peer review only

Appendix 2: Supporting quotes

Sub-theme	Supporting quotes
**Compliance**	
Understanding why supported isolation is necessary	*"I understood the necessity and I was willing to cooperate very much"* (P2)<br>*"I was prepared to [undergo supported isolation] […] I knew it was a necessary evil"* (P19)
Non-compliance through rule-breaking	*"Over a short period of time it was let's try and break the rules just for something to do. Let's see how far we can go"* (P4)
**Feelings about undergoing supported isolation**	
Positive feelings about supported isolation	
Supported isolation protects others	*"we were a risk to others and we didn't want to come back to our families carrying the virus"* (P18)<br>*"it was in our best interests and the people we love in the UK and the country in general"* (P8)
Supported isolation protects self	*"in the event that I or any of my fellow travellers developed symptoms we would be in that hospital environment or we would be with doctors who spoke our native language"* (P9)<br>*"[supported isolation is important] because I would have felt very bad if it had have been found out later that it was from myself that had passed on or started something"* (P11)
Faith in effective management	*"when we actually arrived at Arrowe Park […] the staff there gave such a warm welcome and made everything feel so sort of warm and comfortable"* (P16)
Negative feelings about supported isolation	
Uncertainty about supported isolation	*"You're thinking well what are the facilities here going to be like? How am I going to cope with that?"* (P24)<br>*"I was a little bit apprehensive just because I didn't know […] how it would be structured or organised, and obviously the lack of details"* (P11)
Boredom	*"[I was concerned that] I would be a bit bored"* (P19)
Increased risk of catching COVID-19	*"Our biggest concern would be is anybody sick because of this virus among us?"* (P2)

For peer review only

Thought supported isolation unnecessary	*"we did think it was unnecessary because we were already tested negative"* (P24)
Believed supported isolation a waste of time	*"it was an over the top response that probably cost 2 or 3 million pounds for those two weeks"* (P4)
**Risk perceptions of catching COVID-19**	
Low perceived risk	
Could take protective behaviours	*"we were just very careful with washing our hands […] just sensible hygiene precautions really. So that made us feel pretty safe"* (P16)
Rapid isolation of those with symptoms	*"I knew that things were being monitored very carefully and things were being done about it"* (P4)
Regular testing	*"after one week we'd all been tested negative, after 10 days we'd all been tested negative, after 14 days we'd all been tested negative"* (P4)
High perceived risk	
Others had symptoms	*"someone with a high temperature, she was really close to me, so I said oh please don't stay too close"* (P10)
Close proximity to others	*"we were using the same big meeting room for one or two hours before we eventually went to our separate rooms"* (P2)
Reduced risk perception	*"after the first week we didn't see anyone showing symptoms so everyone was more relaxed talking to each other"* (P10)
**Protective behaviours**	
Stayed in own room	*"we just decided not to go out, just to stay in our hotel rooms"* (P2)
Hand hygiene	*"I would wash my hands when I went downstairs"* (P11)
Wearing a face mask	*"we were wearing gloves and masks and keeping no more contact with each other"* (P3)
**Management of supported isolation**	
Process was well managed	
Process was well-organised	*"the place all sort of ran like clockwork from my point of view"* (P16)<br>*"I don't think they could have done it any better"* (P21)
Process was adapted following feedback	*"the food initially it was only microwave meals but that evolved in the second week […]*

	everybody was sort of learning as we went along" (P5)<br>*"after the first 4 or 5 days everything was up and running and was very smooth and very supportive"* (P12)<br>*"they are improving their responding and they are learning from their mistakes as well they were really good I was really impressed"* (P6)
Process was not well managed	
Meals not received	*"they forgot to give me breakfast and lunch three times"* (P1)
Poor food options	*"when they chose a facility that didn't have fresh food on site they didn't understand the Chinese way of life"* (P8)
Food served uncovered	*"I think most of us had the salad or the bread which was not covered"* (P2)
Food not warm enough	*"the food turned up lukewarm in cardboard boxes"* (P22)
Communication between organisations	*"With the change of shifts, they didn't update people […] there was no passing on of communication, there was no register of requests from room numbers"* (P18)<br>*"there was three branches of the NHS staff there and they all had their own rules […] so it's very difficult to merge as one"* (P4)
**Treatment by supported isolation staff**	
Staff friendly and helpful	*"we were treated with compassion […] and so we were immediately put at ease"* (P24)<br>*"the staff there were really helpful and they were lovely"* (P21)
Staff tried to keep people happy	*"the staff went above and beyond in trying to help us"* (P11)
Staff provided everything people asked for	*"if you needed anything you could get it brought to your room within an hour or two"* (P22)<br>*"staff were very helpful, whatever we asked they tried to answer, and whatever we needed they tried to procure"* (P13)
Staff did not treat people as if they were ill	*"we don't feel that really we were isolated or we were frightening […] as somebody who might carry a virus"* (P2)<br>*"they were actually treating us like normal people"* (P7)
Process did not feel too restrictive	*"I think that's a balance that had to be struck between health risk and […] how we felt that we were being treated, how restricted we felt"* (P11)

For peer review only

**Communication from staff**	
Positive aspects of communication	
Daily newsletter	*"I think they were really good…we would get two or three letters a day actually sometimes about what was changing and why"* (P13)
Proactive communication	*"in the mornings when a nurse would come around […] if there were any sorts of developments to tell us about then they would"* (P15)
Staff answered all questions	*"I think they would have answered anything that we needed to know"* (P22)
Negative aspects of communication	
Staff could not always answer questions	*"the only information [that staff couldn't give me] was sort of about leaving actually, and what was going to happen […] that information was only very near the end"* (P15)
Communication methods old-fashioned	*"their way of disseminating information was posting things under the door, which […] seems a little old-fashioned […] maybe if they had done a group chat or done a group email […] I think that may have been a good way of communicating"* (P16)
Information not provided in multiple languages	*"the Mum […] had to ask for a lot of help because of her difficulties with English, she was a Chinese national"* (P11)
**Communication with those outside isolation**	
Communication with those outside important	*"we spent half the day usually emailing and skyping and WhatsApping everybody […] it was actually good having that routine"* (P24)
Benefit of local community groups	*"it's nice when you are in that situation […] to see stuff that wasn't about the virus, and wasn't doom and gloom"* (P25)
Difficulty communicating with those outside	*"the phone signal where we were was terrible"* (P22)
**Relationship with others**	
People felt a connection	*"I think there was a bit of camaraderie […] everyone was in the same situation really"* (P16)<br>*"we were all in the same boat […] it was just, we were all in it together really"* (P22)
People supported and encouraged each other	*"we look after each other, we tried to be helpful with each other as well"* (P3)
Others' support improved the experience	*"we encouraged each other and things like that sometimes. It was good to help many to spend*

For peer review only

	the long and sometimes worrying days" (P2) *"there was definitely a case of we're going to get through this together"* (P11)
Connection facilitated by chat groups	*"we would message on Facebook and WhatsApp and all that stuff"* (P23) *"we had a little common room within our side of the conference centre […] so we did movie nights and quizzes and things like that"* (P5)
People did not feel a connection	
No opportunity to interact	*"the circumstances didn't really permit interaction"* (P6)
Chose not to interact	*"they all got together and things like that and the invitation was open but at the same time I didn't really want to be in the same room with lots of people"* (P13)
**Perceptions of others' behaviour**	
People trusted others	*"people were very very well-behaved […] people are grateful that was a common feeling"* (P6)
Isolated instances of non-cooperation	*"there's only one argument that we ever heard in the whole two weeks and it was somebody saying that they've been tested negative three times can they go home early […] but apart from that the whole two weeks was like with no issue at all"* (P8)
Most people friendly	*"Almost all […] were quite cooperative [….] I think they were quite friendly to each other"* (P2)
**Areas for improvement**	
More information in advance	*"in advance, it would have been nice to have been told information as to how it works, in terms of freedom of movement and the ability to cook and stuff for yourself […] it's the communication, and setting expectations"* (P25)
Access to outside space	*"outdoor space improvements may have been helpful […] I think we are all finding value in still being able to get outside a little bit"* (P12)
Access to exercise facilities	*"having a couple of running machines and a couple of bikes and things like that would have been really helpful"* (P5)
Improved food	*"there wasn't enough thought put into the requirements of people that were from China […] they didn't understand the Chinese way of life which is very much fresh food based"* (P8)

For peer review only

Feelings after leaving supported isolation	
Felt anxious or overwhelmed	*"I actually had a panic attack when I got in the taxi I found everything very overwhelming […] I hadn't really mentally prepared myself for going outside"* (P23)<br>*"First time we went to the supermarket […] just seeing people who were not in masks and protective clothing took some getting used to. Just being able to wander around and walk […] and all the crowds of people in the supermarket when we'd just been used to us two was quite uncomfortable"* (P24)
Struggled on leaving	*"the last night we were there, there was no sense of jubilation […] it was just very quiet, very subdued. […] [Leaving] affected me quite badly really […] I was absolutely lost"* (P4)<br>*"I stayed at home but I didn't do a great deal except sleep and I was not in the best of moods most of the time…or part of the time"* (P13)
Negative reaction from others	*"the driver who came to pick us up said 'I will have to call head office to get the car disinfected after I drop you off' – that response I think will stay with me for a long time"* (P13)
Follow up information would have been helpful	*"I understand there is a lot happening right now…but I don't think there was enough support for us leaving"* (P23)

For peer review only

BMJ Open

Experiences of supported isolation in returning travellers during the early COVID-19 response: a qualitative interview study

Journal:	*BMJ Open*
Manuscript ID	bmjopen-2021-050405.R1
Article Type:	Original research
Date Submitted by the Author:	13-May-2021
Complete List of Authors:	Carter, Holly; Public Health England Porton, Behavioural Science and Insights Unit<br>Weston, Dale; Public Health England Porton, Emergency Response Department<br>Greenberg , N; King's College London, King's Centre for Military Health Research, Department of Psychological Medicine<br>Oliver, Isabel; Public Health England, Field Epidemiology Services<br>Robin, Charlotte; Public Health England, Field Epidemiology<br>Rubin, GJ; King's College London,<br>Wessely, Simon; Institute of Psychiatry, King's Centre for Military Health Research<br>Gauntlett, Louis; Public Health England Porton<br>Amlot, Richard; Public Health England Porton, Emergency Response Department
**Primary Subject Heading**:	Public health
Secondary Subject Heading:	Qualitative research, Health policy
Keywords:	COVID-19, PUBLIC HEALTH, QUALITATIVE RESEARCH

SCHOLARONE™
Manuscripts

I, the Submitting Author has the right to grant and does grant on behalf of all authors of the Work (as defined in the below author licence), an exclusive licence and/or a non-exclusive licence for contributions from authors who are: i) UK Crown employees; ii) where BMJ has agreed a CC-BY licence shall apply, and/or iii) in accordance with the terms applicable for US Federal Government officers or employees acting as part of their official duties; on a worldwide, perpetual, irrevocable, royalty-free basis to BMJ Publishing Group Ltd ("BMJ") its licensees and where the relevant Journal is co-owned by BMJ to the co-owners of the Journal, to publish the Work in this journal and any other BMJ products and to exploit all rights, as set out in our licence.

The Submitting Author accepts and understands that any supply made under these terms is made by BMJ to the Submitting Author unless you are acting as an employee on behalf of your employer or a postgraduate student of an affiliated institution which is paying any applicable article publishing charge ("APC") for Open Access articles. Where the Submitting Author wishes to make the Work available on an Open Access basis (and intends to pay the relevant APC), the terms of reuse of such Open Access shall be governed by a Creative Commons licence – details of these licences and which Creative Commons licence will apply to this Work are set out in our licence referred to above.

Other than as permitted in any relevant BMJ Author's Self Archiving Policies, I confirm this Work has not been accepted for publication elsewhere, is not being considered for publication elsewhere and does not duplicate material already published. I confirm all authors consent to publication of this Work and authorise the granting of this licence.

For peer review only

Experiences of supported isolation in returning travellers during the early COVID-19 response: a qualitative interview study

Authors: H Carter (0000-0002-2084-7263), D Weston, N Greenberg, I Oliver, C Robin, GJ Rubin, S Wessely, L Gauntlett, R Amlôt.

Behavioural Science and Insights Unit, Public Health England, Porton Down, Salisbury, SP4 0JG, UK

H Carter

Principal Behavioural Scientist

Behavioural Science and Insights Unit, Public Health England, Porton Down, Salisbury, SP4 0JG, UK

D Weston

Principal Behavioural Scientist

Department of Psychological Medicine, King's College London, Weston Education Centre, Cutcombe Road, London, SE5 9RJ, UK

N Greenberg

Professor of Defence Mental Health

National Infection Service, Public Health England, Bristol, UK

I Oliver

Director National Infection Service

Behavioural Science and Insights Unit, Public Health England, Porton Down, Salisbury, SP4 0JG, UK

C Robin

Behavioural Science Team Leader

Department of Psychological Medicine, King's College London, Weston Education Centre, Cutcombe Road, London, SE5 9RJ, UK

GJ Rubin

Reader in the Psychology of Emerging Health Risks

Department of Psychological Medicine, King's College London, Weston Education Centre, Cutcombe Road, London, SE5 9RJ, UK

S Wessely

Professor of Psychological Medicine

Behavioural Science and Insights Unit, Public Health England, Porton Down, Salisbury, SP4 0JG, UK

L Gauntlett

Research Assistant

Behavioural Science and Insights Unit, Public Health England, Porton Down, Salisbury, SP4 0JG, UK R Amlôt

Head of Behavioural Science

For peer review only - http://bmjopen.bmj.com/site/about/guidelines.xhtml

For peer review only

OFFICIAL

Correspondence to: H Carter holly.carter@phe.gov.uk, Behavioural Science and Insights Unit, Public Health England, Porton Down, Salisbury, SP4 0JG, UK.

For peer review only

Abstract

Objectives: 1) to understand the experiences and perceptions of those who underwent supported isolation, particularly in relation to factors that were associated with improved compliance and wellbeing; 2) to inform recommendations for the management of similar supported isolation procedures.

Design: We carried out a qualitative study using semi-structured interviews to capture participants' experiences and perceptions of supported isolation. Data were analysed using the framework approach, a type of thematic analysis that is commonly used in research that has implications for policy.

Setting: Telephone interviews carried out within approximately one month of an individual leaving supported isolation.

Participants: 26 people who underwent supported isolation at either Arrowe Park Hospital (n = 18) or Kents Hill Park Conference Centre (n = 8) after being repatriated from Wuhan in January – February 2020.

Results: Six key themes were identified: factors affecting compliance with supported isolation; risk perceptions around catching COVID-19; management of supported isolation; communication with those outside supported isolation; relationship with others in supported isolation; and feelings on leaving supported isolation. Participants were willing to undergo supported isolation because they understood that it would protect themselves and others. Positive treatment by staff was fundamental to participants' willingness to comply with isolation procedures. Despite the high level of compliance, participants expressed some uncertainty about what the process would involve.

Conclusions: As hotel quarantine is introduced across the UK for international arrivals, our findings suggest that those in charge should: communicate effectively before, during and after quarantine, emphasising why quarantine is important and how it will protect others; avoid coercion if possible and focus on supporting and promoting voluntary compliance; facilitate shared social experiences for those in quarantine; and ensure all necessary supplies are provided. Doing so is likely to increase adherence and reduce any negative effects on wellbeing.

Strengths and limitations of this study

- To our knowledge, the present study is the first research conducted with individuals during and immediately following their supported isolation in the UK as part of the COVID-19 response.
- We used semi-structured interviews to gain an in-depth understanding of the experiences of a sample of people (n = 26) who underwent supported isolation.
- Interviews were carried out within one month of participants leaving supported isolation.
- Our findings are highly topical given the recent introduction of a requirement for travellers to the UK to isolate within hotel accommodation.
- It was not possible to interview everyone who underwent supported isolation, and we were only able to interview those who had a good understanding of English.

For peer review only - http://bmjopen.bmj.com/site/about/guidelines.xhtml

Introduction

The first cases of a novel strain of coronavirus (SARS-CoV-2) were detected in Wuhan, China, in December 2019. On 31st January 2020, British Nationals living in Wuhan were offered repatriation to the UK. 93 returned on two chartered flights. In order to be repatriated all had to agree to undergo 14 days of 'supported isolation'. In some countries and contexts this type of supported isolation is known as quarantine; however, it is typically referred to as supported isolation in the UK, and so will be referred to as supported isolation in the current study. Supported isolation took place in an accommodation block at Arrowe Park Hospital in the Wirral [1], and Kents Hill Park Conference Centre, Milton Keynes. All supported isolation ended by 23rd February 2020 [2]. On arrival at the supported isolation facility, individuals were provided with their own rooms which were fully furnished and had basic cooking, washing and living facilities [3]. Individuals were encouraged to stay in their rooms as much as possible (though this was not mandatory) and could access anything they needed by phoning staff or using an online system; if they did need to leave their rooms they were encouraged to follow hand hygiene guidance and wear a face mask. Individuals also had access to a team of medical staff who closely monitored their condition, including regular testing and symptom checking [3]. There was phone and internet access to enable them to communicate with others both inside and outside the supported isolation facility.

Many countries, including China [4], Vietnam [5], and Singapore [6] have had supported isolation policies in place in response to COVID-19 for over a year, for a variety of situations including international travel. However, supported isolation for returning travellers had, to our knowledge, never been used before within the UK. It was anticipated that the experience could have considerable psychological consequences for the individuals concerned, including potential post-traumatic stress, anger and confusion; consequences that may be affected by a range of stressors including information provision, stigma, and fear of infection [7]. Furthermore, supported isolation represents a unique social context in which relative strangers are placed in close quarters within a novel context and asked to adhere to recommended behaviours for a prolonged period. During emergencies, such social contexts can affect individuals' social identity, which can have consequences for adherence and psychological resilience [8,9,10]. Outside of the emergency response context, the emergence of strong social connections among strangers in close physical proximity has been associated with positive well-being related outcomes [11].

From 15th February 2021 those travelling to the UK from 'red list' countries (countries which have higher prevalence of new COVID-19 variants) [12] have been required to isolate in hotels for 10 days [13]. Countries on the 'red list' are continually reviewed and updated, but as of 9th April 2021 there were 39 countries on the list [14]. Policy around this isolation is focused on identifying the best ways to maximise compliance, with an increasing emphasis on enforcement [15]. Furthermore, with the COVID-19 pandemic ongoing, it is possible that supported isolation will be required in other contexts, such as to assist those with difficulty isolating at home [16] or to reduce household transmission [17]. It is therefore important to understand more about the way in which people experience supported isolation, so that this process can be optimised to increase adherence and mitigate any negative effects on wellbeing. We carried out a rapid mixed-methods study in which we: 1) interviewed individuals who underwent supported isolation at Arrowe Park Hospital and Kents Hill Park conference centre (findings reported here); 2) surveyed those who underwent supported isolation at two time points (immediately after supported isolation and three months after supported isolation) (findings reported elsewhere; Carter et al., in prep). To our knowledge, this is the first research conducted with individuals during and immediately following their supported isolation in this country. With supported isolation now being required for people travelling to the UK from a number of countries, the findings presented here will be invaluable in understanding public experiences of supported isolation and informing optimised management in these settings.

For peer review only - http://bmjopen.bmj.com/site/about/guidelines.xhtml

Aims

This study had two aims: 1) to understand the experiences and perceptions of those who underwent supported isolation, particularly in relation to factors that were associated with improved compliance and wellbeing; 2) to inform the development of recommendations for the management of similar supported isolation procedures.

Method

Ethical approval

Ethical approval was obtained from the Public Health England Research Ethics Governance Group (approval no. NR0187).

Patient and public involvement

Given the extremely rapid and responsive nature of this research, it was not possible to involve patients or the public in the development of the study and associated materials. However, staff at the supported isolation facilities were involved from the outset in planning the study and facilitating participant recruitment. Additionally, findings from this study will be shared with participants on publication.

Design

This study used semi-structured interviews to capture participants' experiences and perceptions of supported isolation. The decision was taken to carry out semi-structured interviews (alongside surveys, reported elsewhere; Carter et al., in prep) in order to generate a more in-depth understanding of participants' perceptions and experiences during supported isolation than could be obtained using surveys alone. Telephone interviews took place within one-month after the isolation. The study was designed and carried out in-line with consolidated criteria for reporting qualitative research (COREQ) guidelines [18] (see Appendix 1).

Participants

Participants underwent supported isolation in either Arrowe Park (n = 18) or Kents Hill Park (n = 8) in January and February 2020. The day before leaving supported isolation, all those in the supported isolation facilities were provided with an information sheet about the study by a member of staff at the facility. This included an invitation to take part in a survey (findings reported elsewhere; Carter et al., in prep), as well as the opportunity to take part in an interview. Thus, voluntary response sampling was used, whereby all those who underwent supported isolation were given the opportunity to take part in both the survey (reported elsewhere; Carter et al., in prep) and an interview, and the sample consisted of those who chose to opt-in to the study. To opt-in to the interview part of the study, participants were asked to provide an email address on leaving supported isolation to enable the research team to follow up and arrange the interview. At this point, 69 people provided a contact email address, and all were then contacted separately and invited to take part in an interview. Of these, 26 people (38%) consented to take part in an interview; this sample therefore represents 12.3% of the entire population who underwent supported isolation.

Materials

An interview schedule was developed to capture in-depth information about individuals' experiences and perceptions of supported isolation, including their: overall experience (e.g. "Tell me about your experience of undergoing supported isolation"); willingness to undergo supported isolation (e.g. "Were you willing to undergo supported isolation"); perceptions of the way the supported isolation process was managed (e.g. "In general, how do you feel the supported isolation process was managed?"); perceptions of others'

For peer review only - http://bmjopen.bmj.com/site/about/guidelines.xhtml

behaviour during supported isolation (e.g. "How did those in supported isolation behave towards each other?"); experiences after leaving supported isolation (e.g. "How has life been for you since leaving supported isolation?"). See Appendix 2 for a copy of the interview schedule.

Procedure

Each interview took place within one month of leaving the supported isolation facility and lasted for approximately an hour. Interviews were carried out by behavioural scientists based at Public Health England or King's College London, all of whom were qualified to at least MSc level and had received training in carrying out interviews. Researchers did not establish a relationship with participants prior to carrying out the interview nor were participants made aware of any personal characteristics of the interviewer, aside from their place of work and the broad aims of the research. Interviews were carried out by both male and female members of the research team. Only the researcher and the participant were present during the interview. Prior to taking part in an interview, participants completed a written consent form. They also provided verbal consent at the start of the interview. Interviews were recorded and subsequently transcribed. After taking part in an interview, participants received a debriefing statement which provided further information about the study, as well as sources of support that participants could access if required.

Analysis

All interviews were completed before beginning data analysis, at which point a framework approach was used to analyse the data [19]. This is a type of thematic analysis that is commonly used within research that has implications for policy and practice [20]. After familiarisation with the data, an initial coding framework was developed based largely on a priori areas of interest in line with the research aims, and specifically included factors that have been shown during previous incidents to be related to compliance and wellbeing.

At this stage, themes were also allowed to emerge from the data. The initial coding framework was intentionally broad, to ensure that areas of interest were not missed, and contained a total of 76 categories, within 22 major themes. The initial framework was discussed with a second researcher, who had also familiarised themselves with the data, and then applied to a small number of transcripts.

The initial coding framework was then refined into an analytical framework, in which codes were grouped together into overarching themes. This resulted in 6 key themes, and 7 sub-themes: factors affecting compliance with supported isolation (two sub-themes: factors promoting compliance; factors threatening compliance);; risk perceptions around catching COVID-19 (two sub-themes: low perceived risk; high perceived risk); management of supported isolation (three sub-themes: operational management; treatment by staff; communication from staff); communication with those outside of supported isolation facilities; relationship with others within supported isolation; feelings on leaving supported isolation. See Table 1 for a full breakdown of themes and sub-themes.

Application of the analytical framework was carried out by hand by the first author, with each passage in the data being coded into one or more of the identified themes. A spreadsheet was used to generate a matrix into which relevant data (e.g. passages of interest relating to each theme) were organised thematically. This enabled data to be compared and contrasted within and between themes and facilitated more in-depth interpretation. After analysing the 26 transcripts no new themes emerged, thus data saturation had been reached [21].

For peer review only - http://bmjopen.bmj.com/site/about/guidelines.xhtml

Table 1: Description of themes and sub-themes

Theme	Sub-theme
Factors affecting compliance	Factors promoting compliance<br>Factors threatening compliance
Risk perceptions around catching COVID-19	Low perceived risk<br>High perceived risk
Management of supported isolation	Operational management<br>Treatment by staff<br>Communication from staff
Communication with those outside	
Relationship with others within supported isolation	
Feelings after leaving supported isolation	

Results

Demographics

Half of the participants (n = 13) were male and half (n = 13) were female. Participants ranged in age from 22 to 78 (mean = 43.2 years). The majority of participants were British nationals (n = 22), with a small number of Chinese nationals (n = 3) and one person who selected 'Other' as their nationality. Similarly, the majority of participants were White British (n = 17), or Chinese (n = 7), with one person being White Irish, and another being Black British. Most participants were educated to degree level or above (n = 17), with a smaller number being educated to higher secondary level (n = 8), and one being educated to primary or lower secondary level. The majority of participants were employed either full time (n = 14) or part time (n = 4). A small number were retired (n = 4), unemployed (n = 2) or self-employed (n = 1), with one participant specifying that they were due to start work following their isolation.

Participants were asked what their reason was for being in Wuhan during the COVID-19 outbreak, and most stated that they were either living there (n = 6), visiting family or friends (n = 8), or on holiday (n = 5). A smaller number were there on a business trip (n = 2), with one participant having been deployed as part of the FCO response. A small number stated that they had not been in Wuhan and were isolating on their return from other affected areas, including Hubei province (n = 2) and the Diamond Princess cruise ship (n = 2). The majority of participants were travelling either with family (n = 11) or on their own (n = 10), with a small number traveling with others they had no relationship with (n = 5). The majority of participants did not share a room (n = 15). Of those that did (n = 11), most shared with family (n = 7) or friends (n = 1), with only a small number sharing with people they didn't know (n = 3).

Focus group discussions

Results are presented by theme below.

Factors affecting compliance with supported isolation

Factors promoting compliance

Most participants were willing to undergo supported isolation. They understood why supported isolation was necessary and why they were being asked to undergo it e.g. "I understood the necessity and I was

For peer review only - http://bmjopen.bmj.com/site/about/guidelines.xhtml

willing to cooperate very much" (KHP2). Most participants felt that the positives of supported isolation outweighed the negatives. Positive aspects were grouped broadly into three themes: a belief that supported isolation protects family and friends as well as UK society e.g. "it was in our best interests and the people we love in the UK and the country in general" (KHP8); a belief that supported isolation would protect themselves, by ensuring they were in a safe place if they developed symptoms and that they would not be blamed in the event of an outbreak in the UK e.g. "in the event that I or any of my fellow travellers developed symptoms we would be in that hospital environment or we would be with doctors who spoke our native language" (AP9); and faith in the effective management of the supported isolation process e.g. "when we actually arrived at Arrowe Park […] the staff there gave such a warm welcome and made everything feel so sort of warm and comfortable" (AP16).

Factors threatening compliance

Where participants expressed concerns these centred around uncertainty about what the process would involve e.g. "You're thinking well what are the facilities here going to be like? How am I going to cope with that?" (AP24), sometimes attributing this to lack of information being provided e.g. "I was a little bit apprehensive just because I didn't know […] how it would be structured or organised, and obviously the lack of details" (AP11). Others were concerned that they would be bored, e.g. "[I was concerned that] I would be a bit bored" (AP19) or would be at increased risk of catching COVID-19, e.g. "Our biggest concern would be is anybody sick because of this virus among us?" (KHP2).

A few felt angry or frustrated about the process, because they didn't think it was necessary e.g. "we did think it was unnecessary because we were already tested negative" (AP24) or believed it was a waste of time and resources e.g. "it was an over the top response that probably cost 2 or 3 million pounds for those two weeks" (KHP4). In the few instances where participants did not want to comply, non-compliance took the form of breaking the rules inside the supported isolation facility (e.g. trying to obtain more alcohol than was allowed), but not trying to leave the supported isolation facility e.g. "Over a short period of time it was let's try and break the rules just for something to do. Let's see how far we can go" (KHP4).

Risk perceptions around catching COVID-19

Low perceived risk

Participants' reported different perceived risks of catching COVID-19 whilst in isolation. Some felt at low risk because they could take protective behaviours e.g. "we were just very careful with washing our hands […] just sensible hygiene precautions really. So that made us feel pretty safe" (AP16). The most commonly reported protective behaviours included staying in their own room e.g. "we just decided not to go out, just to stay in our hotel rooms" (KHP2), observing effective hand hygiene e.g. "I would wash my hands when I went downstairs" (AP11), and wearing a face mask e.g. "we were wearing gloves and masks and keeping no more contact with each other" (KHP3). Other reasons given for low perceptions of risk included that anyone displaying symptoms could be quickly isolated e.g. "I knew that things were being monitored very carefully and things were being done about it" (KHP4), and that everyone in the supported isolation facility underwent regular testing e.g. "after one week we'd all been tested negative, after 10 days we'd all been tested negative, after 14 days we'd all been tested negative" (KHP4).

High perceived risk

However, others were very worried about catching COVID-19 during their stay in supported isolation. Common reasons for this included other people having symptoms e.g. "someone with a high temperature, she was really close to me, so I said oh please don't stay too close" (AP10), and the need to sometimes be in close proximity to others e.g. "we were using the same big meeting room for one or two hours before we

For peer review only - http://bmjopen.bmj.com/site/about/guidelines.xhtml

eventually went to our separate rooms" (KHP2). However, most participants stated that their risk perception reduced over time in the facility, as people continued to test negative, and did not have any symptoms e.g. "towards the end of the isolation, it was getting clearer that nobody in there was probably carrying the virus […] you didn't feel like there was a threat of catching anything from anybody" (AP16). The majority of participants noted that they felt most worried at the start of supported isolation process.

Management of supported isolation

Operational management

Most participants reported that they felt the whole process was well-managed. Reasons for this included that the process was well-organised e.g. "the place all sort of ran like clockwork from my point of view" (AP16), and that staff and management were willing to adapt procedures following negative feedback about the process e.g. "the food initially it was only microwave meals but that evolved in the second week […] everybody was sort of learning as we went along" (KHP5).

Where participants did express concerns these often centred on provision of food, for example not receiving meals e.g. "they forgot to give me breakfast and lunch three times" (KHP1), food being served uncovered e.g. "I think most of us had the salad or the bread which was not covered" (KHP2), and food not being warm enough e.g. "the food turned up lukewarm in cardboard boxes" (AP22). Relatedly, several participants felt that the cultural background of those undergoing supported isolation had not been properly considered. For example, many travellers were Chinese nationals and fresh food is very important to people in China e.g. "when they chose a facility that didn't have fresh food on site they didn't understand the Chinese way of life" (KHP8); the ready meals and pre-prepared foods provided in the first few days of supported isolation were therefore inappropriate.

Another area of management that participants suggested could be improved was around internal communication within and between organisations e.g. "With the change of shifts, they didn't update people […] there was no passing on of communication, there was no register of requests from room numbers (AP18). A final consideration raised in relation to operational management of supported isolation was that several participants would have liked more access to outside space and exercise facilities e.g. "outdoor space improvements may have been helpful […] I think we are all finding value in still being able to get outside a little bit (AP12). For the most part, participants who provided negative feedback about the operational management of the supported isolation process felt that changes were made to address their concerns, and that the management of the supported isolation process improved as time went on e.g. "they are improving their responding and they are learning from their mistakes as well they were really good I was really impressed" (KHP6).

Treatment by supported isolation staff and authorities

Overall, participants were extremely positive in their feedback about the way in which staff treated them. The staff were friendly and helpful e.g. "we were treated with compassion […] and so we were immediately put at ease" (AP24), went out of their way to keep people happy e.g. "the staff went above and beyond in trying to help us" (AP11), and provided people with anything that they asked for e.g. "staff were very helpful, whatever we asked they tried to answer, and whatever we needed they tried to procure" (AP13). A few participants mentioned that staff did not try to avoid them or treat them as if they were ill e.g. "we don't feel that really we were isolated or we were frightening […] as somebody who might carry a virus" (KHP2). A small number of participants specifically noted that staff achieved a good balance between promoting good public health, without making the process too restrictive e.g. "I think that's a balance that had to be struck between health risk and […] how we felt that we were being treated, how restricted we felt" (AP11).

For peer review only - http://bmjopen.bmj.com/site/about/guidelines.xhtml

Communication from staff during supported isolation

Participants were also overwhelmingly positive about the way in which members of staff communicated with them. Almost all participants talked about the daily newsletter that they received from staff and felt that this was an effective way of providing information about protective actions, timings of any activities, and testing e.g. "I think they were really good…we would get two or three letters a day actually sometimes about what was changing and why" (AP13). Similarly, participants noted that staff were proactive in their communications, calling regularly to check on each individual e.g. "in the mornings when a nurse would come around […] if there were any sorts of developments to tell us about then they would" (AP15), and scheduling regular update meetings. Participants also felt that staff answered all their questions (or tried to) and were open and transparent in providing information e.g. "I think they would have answered anything that we needed to know" (AP22).

Some expressed dissatisfaction at the somewhat old-fashioned methods of communication e.g. "their way of disseminating information was posting things under the door, which […] seems a little old-fashioned […] maybe if they had done a group chat or done a group email […] I think that may have been a good way of communicating" (AP16), and information not being provided in multiple languages e.g. "the Mum […] had to ask for a lot of help because of her difficulties with English, she was a Chinese national" (AP11). A small number of participants also felt that staff had been unable to answer some questions e.g. "the only information [that staff couldn't give me] was sort of about leaving actually, and what was going to happen […] that information was only very near the end" (AP15).

Communication with those outside of supported isolation facilities

Most participants found it easy to communicate with those outside supported isolation and did so regularly. Several participants expressed how important this was in helping them to get through the supported isolation process e.g. "we spent half the day usually emailing and skyping and WhatsApping everybody […] it was actually good having that routine" (AP24). Additionally, some were able to carry on working during supported isolation, and this helped them to pass the time. A few participants also highlighted the benefit of local community groups who posted pictures of uplifting things e.g. "it's nice when you are in that situation […] to see stuff that wasn't about the virus, and wasn't doom and gloom" (AP25).

On the other hand, some participants did note difficulties in communicating with those outside of supported isolation, and these typically related to having limited access to internet or poor phone signal e.g. "the phone signal where we were was terrible" (AP22).

Relationship with others within supported isolation

Where people felt a connection with others this was often due to a sense of camaraderie e.g. "I think there was a bit of camaraderie […] everyone was in the same situation really" (AP16) or shared experience e.g. "we were all in the same boat […] it was just, we were all in it together really" (AP22). Some participants described how people supported and encouraged each other during the supported isolation process e.g. "we look after each other, we tried to be helpful with each other as well" (KHP3), stating that this helped people to get through the experience e.g. "we encouraged each other and things like that sometimes. It was good to help many to spend the long and sometimes worrying days" (KHP2). This connection was facilitated by the formation of chat groups e.g. "we would message on Facebook and WhatsApp and all that stuff" (AP23), and some level of freedom to socialise with others e.g. "we had a little common room within our side of the conference centre […] so we did movie nights and quizzes and things like that" (KHP5).

Where people did not feel a connection with others this was because they either didn't get the opportunity to interact much with others, or actively avoided it (due to fears about catching COVID-19) e.g. "they all got

For peer review only - http://bmjopen.bmj.com/site/about/guidelines.xhtml

together and things like that and the invitation was open but at the same time I didn't really want to be in the same room with lots of people" (AP13).

Most participants felt that they could trust others to behave appropriately and instances of uncooperative behaviour were rare or non-existent e.g. "people were very very well-behaved […] people are grateful that was a common feeling" (KHP6). A handful of participants noted isolated instances of uncooperative behaviour e.g. "there's only one argument that we ever heard in the whole two weeks and it was somebody saying that they've been tested negative three times can they go home early […] but apart from that the whole two weeks was like with no issue at all (KHP8), but almost all said that the majority of people were friendly and cooperative "Almost all […] were quite cooperative […] I think they were quite friendly to each other" (KHP2).

Feelings after leaving supported isolation

Many participants felt happy and relieved to leave supported isolation and get back to normal e.g. "I've never been so happy to see my own bed […] and my own house" (AP16). Most people felt that others had treated them normally on leaving supported isolation, and that they hadn't experienced negative reactions from others e.g. "nobody has reacted any different to me" (AP17).

However, several participants stated that they struggled after leaving supported isolation. Some felt anxious or overwhelmed, with reasons including not being used to going outside e.g. "I actually had a panic attack when I got in the taxi I found everything very overwhelming […] I hadn't really mentally prepared myself for going outside" (AP23), or being concerned about mixing with large numbers of people again e.g. "First time we went to the supermarket […] just seeing people who were not in masks and protective clothing took some getting used to […] all the crowds of people in the supermarket when we'd just been used to us two was quite uncomfortable" (AP24). Others simply stated that they had generally struggled on leaving e.g. "the last night we were there, there was no sense of jubilation […] it was just very quiet, very subdued. [Leaving] affected me quite badly really […] I was absolutely lost" (KHP4), or that they had experienced negative reactions from others e.g. "the driver who came to pick us up said 'I will have to call head office to get the car disinfected after I drop you off' – that response I think will stay with me for a long time" (AP13).

The majority of participants did not receive follow up information, though a few did receive information about sources of further support. While some stated that they would not have expected to receive any additional information, others felt that this would have been helpful e.g. "I understand there is a lot happening right now…but I don't think there was enough support for us leaving" (AP23).

Discussion

This paper is the first in-depth analysis of the experiences of those who underwent supported isolation in the UK during the first wave of the COVID-19 pandemic. The findings therefore provide a unique insight into the way in which members of the public perceive supported isolation in the UK, and the factors that affect compliance and wellbeing in such settings. Given that supported isolation is once again required in the management of COVID-19 in the UK [12,13,15], our findings should help facilitate optimised management of supported isolation procedures.

Despite some initial concerns, including confusion about what the process would involve and fears of infection, all willingly complied with the voluntary supported isolation process. People understood why it was necessary and believed that doing so would protect themselves, their friends and family, and others in the UK; motivation for adherence was largely altruistic. Participants were overwhelmingly positive about their treatment by staff, communication from staff, and overall management of the supported isolation process. This was fundamental to participants' willingness to comply with the restrictions of their liberty.

For peer review only - http://bmjopen.bmj.com/site/about/guidelines.xhtml

Our findings are in line with systematic reviews carried out at the start of the pandemic [7,22], as well as research into the management of other types of emergencies [8,23]. Crucially, participants believed their treatment by staff was legitimate, and they therefore chose to comply with supported isolation procedures; it is likely that compliance would have been much lower had staff attempted to enforce compliance [23].

There were mixed views as to whether people in isolation experienced a connection with each other. However, almost all reported that others were helpful and friendly. Additionally, a number of people developed a shared identity with others; for example, they talked about everyone being in it together or going through the same experience. Those that did develop a shared identity often reported that this helped them to get through the process. This is as would be expected based on previous research which suggests that when people experience a sense of shared identity with others, this promotes adherence to protective measures, resilience and well-being [8,9,11]. While a sense of shared social identity arose spontaneously in some instances, participants emphasised that being able to communicate with others (for example, via chat groups) enhanced the social support that they experienced. Promoting virtual interaction between those undergoing supported isolation may be beneficial for strengthening shared identity, facilitating provision of social support, and promoting resilience and well-being. Further research could examine how best to employ virtual methods (e.g. WhatsApp groups, social media) to foster shared social identity and social support amongst those undergoing supported isolation, and the impact that this might have on experiences and behaviours during supported isolation. Participants also highlighted how important it was that they were able to easily keep in touch with friends and family during the supported isolation process.

While most participants reported either positive or neutral experiences during supported isolation, it was interesting to note that some reported negative experiences on leaving supported isolation. Findings suggest that it may be beneficial to prepare those undergoing supported isolation for possible psychosocial reactions they may experience upon leaving supported isolation (e.g. feeling anxious or overwhelmed), assist them with logistical aspects associated with leaving supported isolation (e.g. organising travel home, contacting loved ones), and signpost them to sources of support. These would address many of the negative experiences upon leaving supported isolation. However, some participants stated that they had struggled on leaving supported isolation but were not able to explain why that was the case. Further research should be carried out to better understand why some individuals may struggle on leaving supported isolation and improve support to these individuals.

The supported isolation carried out in January – February 2020 was designed to support those who were returning to the UK, and every effort was made to ensure that their experience was as positive as possible; as participants noted, staff could not do enough for them. Isolation in hotels is likely to be very different, with limited support from staff and an emphasis increasingly on enforcement rather than encouragement [15]. The reasons why people are travelling in the middle of a pandemic will also be different. The UK may find itself placing people into isolation who are more likely to experience distress such as those who are arriving to attend a funeral, are travelling due to a family crisis, or who do not speak English. We must also not forget that, unlike travellers placed into facilities at Arrowe Park or Kents Hill, returning travellers will now be asked to pay £1,500 each towards their isolation.

It is therefore critical that those responsible for implementing policies on isolation requirements take into account the recommendations presented here; failure to do so is likely to reduce adherence to isolation and risks serious long-term impact on those involved. Further research should explore travellers' experiences of undergoing supported isolation within one of the designated hotels. Due to the key differences (outlined above) between these hotels and the supported isolation reported in this paper, this should be compared with the experiences of those who underwent supported isolation at Arrowe Park of Kents Hill Park, and further our understanding of factors affecting compliance and wellbeing in supported isolation settings.

For peer review only - http://bmjopen.bmj.com/site/about/guidelines.xhtml

Limitations

We have no information on those who did not participate, and it is possible that they differed on key variables. Of those that did, we reached thematic saturation within the sample. A second limitation is that only those who had a good understanding of English were interviewed. It is possible that the experience differed for those who were less able to understand English; indeed, this was alluded to in some comments made by participants. A final limitation is that this study was jointly run by King's College London and Public Health England, and Public Health England also assisted with the management of the supported isolation process. The team carrying out this research were not associated with the management of the supported isolation process, although did provide advice to the teams involved. It is therefore possible that participants were aware that PHE played a role in managing the supported isolation process.

Conclusion and Recommendations

Our findings, viewed in the context of the wider relevant published literature, generate several key recommendations that are particularly relevant given the upcoming requirement for travellers to isolate in hotels. Specific recommendations are: 1) prior to supported isolation, authorities should communicate with those affected about why isolation is necessary, how it will help to protect others, and what the process will involve. Given that compliance is often motivated by altruism, emphasising how isolation will protect others is crucial. Such communication will also reduce concerns related to uncertainty about the isolation process; 2) authorities should communicate effectively with those undergoing isolation, throughout the process. Communication should be open and honest, and information should include protective actions people should take, why taking such actions is effective, and how taking such actions protects oneself and others; 3) enforcement of isolation should be avoided wherever possible. Given the large numbers of people who may be required to isolate at one time it will not be possible to enforce adherence; attempting to do so is likely to be perceived as illegitimate, thereby reducing adherence and risking serious long term consequences for those involved; 4) it is likely to be helpful to facilitate and encourage development of shared identity among those undergoing supported isolation, via the formation of chat groups or other means of communication, that include staff managing the facilities. This type of shared social identity should encourage both adherence to supported isolation measures, and improved resilience during the supported isolation process; 5) it is important to ensure that all essential supplies (such as food, exercise facilities, ability to communicate with those outside isolation) are provided and are suitable for the needs of the traveller; 6) authorities should provide relevant information prior to leaving supported isolation to help people to prepare to return to their normal lives. Relevant information should cover the emotions that people might experience, and sources of further support that people can access if required. It may also be beneficial to include in this information any ongoing expectations around adherence to protective behaviours.

For peer review only - http://bmjopen.bmj.com/site/about/guidelines.xhtml

Competing interest statement

All authors have completed the ICMJE uniform disclosure form and declare: HC, DW, IO, CR, and RA are current employees of Public Health England; GJR participates in the UK's Scientific Advisory Group for Emergencies and its subgroups.

Transparency declaration

The lead author affirms that this manuscript is an honest, accurate, and transparent account of the study being reported; that no important aspects of the study have been omitted; and that any discrepancies from the study as originally planned (and, if relevant, registered) have been explained.

Contributor statement

RA, HC, DW, NG, GJR, IO and SW conceived the study. HC, CR, DW and RA collected the data. HC and LG carried out data familiarisation and developed the coding framework. HC carried out the analysis and wrote the first draft of the manuscript. All authors contributed to the design and implementation of the study, and to the writing of the manuscript.

Data availability statement

Data are available on request from holly.carter@phe.gov.uk.

Funding statement

This study was funded by the National Institute for Health Research Health Protection Research Unit (NIHR HPRU) in Emergency Preparedness and Response (grant number 200890), a partnership between Public Health England, King's College London and the University of East Anglia. DW, IO, CR and RA are supported by the NIHR HPRU in Behavioural Science and Evaluation, a partnership between Public Health England and the University of Bristol. CR is also supported by the NIHR HPRU in Emerging and Zoonotic Infections and NIHR HPRU in Gastrointestinal Infections. The views expressed are those of the authors and not necessarily those of the NIHR, Public Health England or the Department of Health and Social Care.

For peer review only

For peer review only - http://bmjopen.bmj.com/site/about/guidelines.xhtml

References

1. BBC News. *Coronavirus: Britons evacuated from Wuhan arrive at quarantine facility*. BBC News [newspaper on the Internet] 2020 Jan 31 [cited 2021 Feb 2]. Available from: https://www.bbc.co.uk/news/uk-51318691.

2. BBC News. *Coronavirus: Wuhan Britons end quarantine as cruise passengers isolate*. BBC News [newspaper on the Internet] 2020 Feb 23 [cited 2021 Feb 2]. Available from: https://www.bbc.co.uk/news/uk-51602986.

3. ITV News. *Britons in quarantine after Wuhan evacuation as death toll rises*. ITV News [news website] 2020 Jan 31 [cited 2021 Apr 26]. Available from: https://www.itv.com/news/2020-01-31/evacuated-britons-arrive-at-wirral-hospital-to-begin-fortnight-in-quarantine

4. Li X, Liu M, Zhou R, Zhang Y, Wu C, Xu, L. Centralized medical quarantine for imported COVID-19 in Shanghai, China. *J Travel Med* 2020;27(5):109.

5. Pollack T, Thwaites G, Rabaa M, et al. *Emerging COVID-19 success story: Vietnam's commitment to containment*. Our World in Data 2021 Mar 5 [cited 2021 Apr 26]. Available from: https://ourworldindata.org/covid-exemplar-vietnam

6. Chia ML, Chau DHH, Lim KS, Liu CWY, Tan HK, Tan YR. Managing COVID-19 in a novel, rapidly deployable community isolation quarantine facility. *Annals of Internal Medicine* 2021.

7. Brooks SK, Webster R, Smith LE, et al. The psychological impact of quarantine and how to reduce it: Rapid review of the evidence. *The Lancet* 2020;395(10227):912–920.

8. Carter H, Drury J, Rubin GJ, Williams R, Amlôt R. Applying crowd psychology to develop recommendations for the management of mass decontamination. *Health Security* 2015;13(1):45–53.

9. Drury J, Carter H, Cocking C, Ntontis E, Tekin Guven S, Amlôt R. Facilitating collective psychosocial resilience in the public in emergencies: twelve recommendations based on the social identity approach. *Frontiers in Public Health* 2019;7(141):Doi: 10.3389/fpubh.2019.00141.

10. Drury J, Carter H, Ntontis E, Tekin Guven S. Public behaviour in response to the COVID-19 pandemic: understanding the role of group processes. *BJPsych Open* 2020;1:1–6.

11. Howell JL, Koudenberg N, Loschelder DD, et al. Happy but unhealthy: The relationship between social ties and health in an emerging network. *European Journal of Social Psychology* 2014;44(6):612–621.

12. Department of Health and Social Care. *Booking and staying in a quarantine facility when you arrive in England*. Department of Health and Social Care 2021 Apr 23 [cited 2021 Apr 26]. Available from: https://www.gov.uk/guidance/booking-and-staying-in-a-quarantine-hotel-when-you-arrive-in-england#who-has-to-go-into-managed-quarantine-in-a-hotel

13. BBC News. *Covid: Quarantine hotel plans set to be announced*. BBC News [newspaper on the Internet] 2020 Jan 26 [cited 2021 Feb 2]. Available from: https://www.bbc.co.uk/news/uk-politics-55805575.

14. Department for Transport. *Coronavirus (COVID-19): red list travel ban countries*. Department for Transport 2021 Apr 23 [cited 2021 Apr 26]. Available from: https://www.gov.uk/guidance/transport-measures-to-protect-the-uk-from-variant-strains-of-covid-19

For peer review only - http://bmjopen.bmj.com/site/about/guidelines.xhtml

15. BBC News. *Covid-19: Travellers face £1,750 cost for England quarantine hotels*. BBC News [newspaper on the Internet] 2021 Feb 9 [cited 2021 Feb 9]. Available from: https://www.bbc.co.uk/news/uk-55995645 9th February 2021.

16. Gurdasani D, Bear L, Bogaert D, et al. The UK needs a sustainable strategy for COVID-19. *The Lancet* 2020;396:1800–1801.

17. Denford S, Morton K, Horwood J, de Garang R, Yardley L. Preventing within household transmission of COVID-19: Is the provision of accommodation feasible and acceptable? *medRxiv* 2020.

18. Tong A, Sainsbury P, Craig J. Consolidated criteria for reporting qualitative research (COREQ): a 32-item checklist for interviews and focus groups. *International Journal for Quality in Health Care* 2007;19(6):349-357.

19. Ritchie J, Spencer L. Qualitative data analysis for applied policy research. In: Bryman A, Burgess RG, eds. *Analyzing qualitative data*. London, UK: Routledge 1994. 173 – 194.

20. Pope C, Ziebland S, Mays N. Analysing qualitative data. *BMJ* 2000;320:114–116.

21. Glaser BG, Strauss AL. *The discovery of grounded theory: strategies for qualitative theory*. New Brunswick: Aldine Transaction; 1967.

22. Webster RK, Brooks SK, Smith L, Woodland L, Wessely S, Rubin GJ. How to improve adherence with quarantine: Rapid review of the evidence. *Public Health* 2020;182:163–169.

23. Carter H, Drury J, Amlôt R. Social identity and intergroup relationships in the management of crowds during mass emergencies and disasters: recommendations for emergency planners and responders. *Policing: A Journal of Policy and Practice* 2018:1–14.

For peer review only

For peer review only - http://bmjopen.bmj.com/site/about/guidelines.xhtml

COREQ (COnsolidated criteria for REporting Qualitative research) Checklist

A checklist of items that should be included in reports of qualitative research. You must report the page number in your manuscript where you consider each of the items listed in this checklist. If you have not included this information, either revise your manuscript accordingly before submitting or note N/A.

Topic	Item No.	Guide Questions/Description	Reported on Page No.
**Domain 1: Research team and reflexivity**			
Personal characteristics			
Interviewer/facilitator	1	Which author/s conducted the interview or focus group?	
Credentials	2	What were the researcher's credentials? E.g. PhD, MD	
Occupation	3	What was their occupation at the time of the study?	
Gender	4	Was the researcher male or female?	
Experience and training	5	What experience or training did the researcher have?	
Relationship with participants			
Relationship established	6	Was a relationship established prior to study commencement?	
Participant knowledge of the interviewer	7	What did the participants know about the researcher? e.g. personal goals, reasons for doing the research	
Interviewer characteristics	8	What characteristics were reported about the inter viewer/facilitator? e.g. Bias, assumptions, reasons and interests in the research topic	
**Domain 2: Study design**			
Theoretical framework			
Methodological orientation and Theory	9	What methodological orientation was stated to underpin the study? e.g. grounded theory, discourse analysis, ethnography, phenomenology, content analysis	
Participant selection			
Sampling	10	How were participants selected? e.g. purposive, convenience, consecutive, snowball	
Method of approach	11	How were participants approached? e.g. face-to-face, telephone, mail, email	
Sample size	12	How many participants were in the study?	
Non-participation	13	How many people refused to participate or dropped out? Reasons?	
Setting			
Setting of data collection	14	Where was the data collected? e.g. home, clinic, workplace	
Presence of non-participants	15	Was anyone else present besides the participants and researchers?	
Description of sample	16	What are the important characteristics of the sample? e.g. demographic data, date	
Data collection			
Interview guide	17	Were questions, prompts, guides provided by the authors? Was it pilot tested?	
Repeat interviews	18	Were repeat inter views carried out? If yes, how many?	
Audio/visual recording	19	Did the research use audio or visual recording to collect the data?	
Field notes	20	Were field notes made during and/or after the inter view or focus group?	
Duration	21	What was the duration of the inter views or focus group?	
Data saturation	22	Was data saturation discussed?	
Transcripts returned	23	Were transcripts returned to participants for comment and/or	

For peer review only

Topic	Item No.	Guide Questions/Description	Reported on Page No.
		correction?	
**Domain 3: analysis and findings**			
Data analysis			
Number of data coders	24	How many data coders coded the data?	
Description of the coding tree	25	Did authors provide a description of the coding tree?	
Derivation of themes	26	Were themes identified in advance or derived from the data?	
Software	27	What software, if applicable, was used to manage the data?	
Participant checking	28	Did participants provide feedback on the findings?	
Reporting			
Quotations presented	29	Were participant quotations presented to illustrate the themes/findings? Was each quotation identified? e.g. participant number	
Data and findings consistent	30	Was there consistency between the data presented and the findings?	
Clarity of major themes	31	Were major themes clearly presented in the findings?	
Clarity of minor themes	32	Is there a description of diverse cases or discussion of minor themes?	

Developed from: Tong A, Sainsbury P, Craig J. Consolidated criteria for reporting qualitative research (COREQ): a 32-item checklist for interviews and focus groups. *International Journal for Quality in Health Care*. 2007. Volume 19, Number 6: pp. 349 – 357

Once you have completed this checklist, please save a copy and upload it as part of your submission. DO NOT include this checklist as part of the main manuscript document. It must be uploaded as a separate file.

For peer review only

Appendix 2: Topic guide with questions relating to participants' experiences during and after supported isolation

During supported isolation

Where did you undergo supported isolation?

- Prompts:
    - If you underwent supported isolation at Arrowe Park, what date did you leave?

What were your thoughts about undergoing supported isolation?

- Prompts:
    - Did you understand why you were being put into supported isolation?
    - Did you have any concerns about undergoing supported isolation?
    - If so what were your concerns?
    - Did you think there were any benefits to you of undergoing supported isolation?

Tell me about your experience of undergoing supported isolation.

- Prompts:
    - What has it involved?
    - How has it been?

Were you aware of anyone who was in supported isolation with you having symptoms/ having the coronavirus?

- Prompts:
    - If so, what did you think/ how did you react?

In general, how do you feel the supported isolation process was managed at Arrowe Park/ Kents Hill Park?

Did you feel that staff/ authorities treated you fairly whilst you were in supported isolation?

- Prompts:
    - If so, why?
    - If not, why?

How did you feel about staff/ authority ability to successfully manage the supported isolation process?

Did you trust that the supported isolation process was being managed effectively?

Was there anything that could have been done to improve the way the supported isolation process was managed?

For peer review only

How well do you think staff/ authorities communicated with you whilst you were at Arrowe Park/ Kents Hill Park?

Did staff answer any questions you had?

Did you feel that you were provided with information that you needed?

- Prompts:
    - Were you provided with information about the incident?
    - Were you provided with information about what actions you should take?
    - Were you provided with information about why you were being asked to take certain actions?
    - Was there information you would have liked/ needed that you didn't receive? If so, what?

Did you get the opportunity to communicate with anyone outside of the supported isolation facility?

- Prompts:
    - If so, with who?
    - If so, how did you communicate with them (e.g. phone, social media, email etc)?
    - If so, how often?

Were you willing to undergo supported isolation?

- Prompts:
    - If so why?
    - If not why?
    - If not, what would have made it more likely you would comply?

How do you feel towards the other people who were in supported isolation with you?

- Prompts:
    - Did you feel any connection with them (e.g. bond, shared fate etc)
    - Were you concerned about the possibility of being infected by others?

Did you spend much time with them/ interact much with them?

Did your feelings about the other people affected change over the course of your time in supported isolation?

How did those in supported isolation behave towards each other?

Did you trust that other individuals in supported isolation would behave appropriately?

For peer review only

Did you see anyone refuse to undergo supported isolation?

- Prompts:
    - If so, what action did authorities take?
    - How did other people react?

Post-supported isolation

How has life been for you since leaving supported isolation?

Have you been given follow up information?

Overall, what do you think about the way the Government is managing this outbreak?

Do you feel you have experienced any unhelpful responses by others since undergoing supported isolation?

- Prompts:
    - Do you feel that people have reacted differently to you as a result of your having undergone supported isolation?
    - Do you feel that people have avoided you as a result of your having undergone supported isolation?

Is there anything else you'd like to say that hasn't been covered here?

For peer review only

BMJ Open

Experiences of supported isolation in returning travellers during the early COVID-19 response: a qualitative interview study

Journal:	*BMJ Open*
Manuscript ID	bmjopen-2021-050405.R2
Article Type:	Original research
Date Submitted by the Author:	18-Jun-2021
Complete List of Authors:	Carter, Holly; Public Health England Porton, Behavioural Science and Insights Unit<br>Weston, Dale; Public Health England Porton, Emergency Response Department<br>Greenberg , N; King's College London, King's Centre for Military Health Research, Department of Psychological Medicine<br>Oliver, Isabel; Public Health England, Field Epidemiology Services<br>Robin, Charlotte; Public Health England, Field Epidemiology<br>Rubin, GJ; King's College London,<br>Wessely, Simon; Institute of Psychiatry, King's Centre for Military Health Research<br>Gauntlett, Louis; Public Health England Porton<br>Amlot, Richard; Public Health England Porton, Emergency Response Department
**Primary Subject Heading**:	Public health
Secondary Subject Heading:	Qualitative research, Health policy
Keywords:	COVID-19, PUBLIC HEALTH, QUALITATIVE RESEARCH

SCHOLARONE™
Manuscripts

I, the Submitting Author has the right to grant and does grant on behalf of all authors of the Work (as defined in the below author licence), an exclusive licence and/or a non-exclusive licence for contributions from authors who are: i) UK Crown employees; ii) where BMJ has agreed a CC-BY licence shall apply, and/or iii) in accordance with the terms applicable for US Federal Government officers or employees acting as part of their official duties; on a worldwide, perpetual, irrevocable, royalty-free basis to BMJ Publishing Group Ltd ("BMJ") its licensees and where the relevant Journal is co-owned by BMJ to the co-owners of the Journal, to publish the Work in this journal and any other BMJ products and to exploit all rights, as set out in our licence.

The Submitting Author accepts and understands that any supply made under these terms is made by BMJ to the Submitting Author unless you are acting as an employee on behalf of your employer or a postgraduate student of an affiliated institution which is paying any applicable article publishing charge ("APC") for Open Access articles. Where the Submitting Author wishes to make the Work available on an Open Access basis (and intends to pay the relevant APC), the terms of reuse of such Open Access shall be governed by a Creative Commons licence – details of these licences and which Creative Commons licence will apply to this Work are set out in our licence referred to above.

Other than as permitted in any relevant BMJ Author's Self Archiving Policies, I confirm this Work has not been accepted for publication elsewhere, is not being considered for publication elsewhere and does not duplicate material already published. I confirm all authors consent to publication of this Work and authorise the granting of this licence.

For peer review only

Experiences of supported isolation in returning travellers during the early COVID-19 response: a qualitative interview study

Authors: H Carter (0000-0002-2084-7263), D Weston, N Greenberg, I Oliver, C Robin, GJ Rubin, S Wessely, L Gauntlett, R Amlôt.

H Carter

Principal Behavioural Scientist

Behavioural Science and Insights Unit, Public Health England, Porton Down, Salisbury, SP4 0JG, UK

D Weston

Principal Behavioural Scientist

Behavioural Science and Insights Unit, Public Health England, Porton Down, Salisbury, SP4 0JG, UK

N Greenberg

Professor of Defence Mental Health

Department of Psychological Medicine, King's College London, Weston Education Centre, Cutcombe Road, London, SE5 9RJ, UK

I Oliver

Director National Infection Service

National Infection Service, Public Health England, Bristol, UK

C Robin

Behavioural Science Team Leader

Behavioural Science and Insights Unit, Public Health England, Porton Down, Salisbury, SP4 0JG, UK

GJ Rubin

Reader in the Psychology of Emerging Health Risks

Department of Psychological Medicine, King's College London, Weston Education Centre, Cutcombe Road, London, SE5 9RJ, UK

S Wessely

Professor of Psychological Medicine

Department of Psychological Medicine, King's College London, Weston Education Centre, Cutcombe Road, London, SE5 9RJ, UK

L Gauntlett

Research Assistant

Behavioural Science and Insights Unit, Public Health England, Porton Down, Salisbury, SP4 0JG, UK

R Amlôt

Head of Behavioural Science

For peer review only

OFFICIAL

Behavioural Science and Insights Unit, Public Health England, Porton Down, Salisbury, SP4 0JG, UK

Correspondence to: H Carter holly.carter@phe.gov.uk, Behavioural Science and Insights Unit, Public Health England, Porton Down, Salisbury, SP4 0JG, UK.

For peer review only

Abstract

Objectives: 1) to understand the experiences and perceptions of those who underwent supported isolation, particularly in relation to factors that were associated with improved compliance and wellbeing; 2) to inform recommendations for the management of similar supported isolation procedures.

Design: We carried out a qualitative study using semi-structured interviews to capture participants' experiences and perceptions of supported isolation. Data were analysed using the framework approach, a type of thematic analysis that is commonly used in research that has implications for policy.

Setting: Telephone interviews carried out within approximately one month of an individual leaving supported isolation.

Participants: 26 people who underwent supported isolation at either Arrowe Park Hospital (n = 18) or Kents Hill Park Conference Centre (n = 8) after being repatriated from Wuhan in January – February 2020.

Results: Six key themes were identified: factors affecting compliance with supported isolation; risk perceptions around catching COVID-19; management of supported isolation; communication with those outside supported isolation; relationship with others in supported isolation; and feelings on leaving supported isolation. Participants were willing to undergo supported isolation because they understood that it would protect themselves and others. Positive treatment by staff was fundamental to participants' willingness to comply with isolation procedures. Despite the high level of compliance, participants expressed some uncertainty about what the process would involve.

Conclusions: As hotel quarantine is introduced across the UK for international arrivals, our findings suggest that those in charge should: communicate effectively before, during and after quarantine, emphasising why quarantine is important and how it will protect others; avoid coercion if possible and focus on supporting and promoting voluntary compliance; facilitate shared social experiences for those in quarantine; and ensure all necessary supplies are provided. Doing so is likely to increase adherence and reduce any negative effects on wellbeing.

Strengths and limitations of this study

- To our knowledge, the present study is the first research conducted with individuals during and immediately following their supported isolation in the UK as part of the COVID-19 response.
- We used semi-structured interviews to gain an in-depth understanding of the experiences of a sample of people (n = 26) who underwent supported isolation.
- Interviews were carried out within one month of participants leaving supported isolation.
- Our findings are highly topical given the recent introduction of a requirement for travellers to the UK to isolate within hotel accommodation.
- It was not possible to interview everyone who underwent supported isolation, and we were only able to interview those who had a good understanding of English.

For peer review only - http://bmjopen.bmj.com/site/about/guidelines.xhtml

For peer review only

Introduction

The first cases of a novel strain of coronavirus (SARS-CoV-2) were detected in Wuhan, China, in December 2019. On 31st January 2020, British Nationals living in Wuhan were offered repatriation to the UK. 93 returned on two chartered flights. In order to be repatriated all had to agree to undergo 14 days of 'supported isolation'. In some countries and contexts this type of supported isolation is known as quarantine; however, it is typically referred to as supported isolation in the UK, and so will be referred to as supported isolation in the current study. Supported isolation took place in an accommodation block at Arrowe Park Hospital in the Wirral [1], and Kents Hill Park Conference Centre, Milton Keynes. All supported isolation ended by 23rd February 2020 [2]. On arrival at the supported isolation facility, individuals were provided with their own rooms which were fully furnished and had basic cooking, washing and living facilities [3]. Individuals were encouraged to stay in their rooms as much as possible (though this was not mandatory) and could access anything they needed by phoning staff or using an online system; if they did need to leave their rooms they were encouraged to follow hand hygiene guidance and wear a face mask. Individuals also had access to a team of medical staff who closely monitored their condition, including regular testing and symptom checking [3]. There was phone and internet access to enable them to communicate with others both inside and outside the supported isolation facility.

Many countries, including China [4], Vietnam [5], and Singapore [6] have had supported isolation policies in place in response to COVID-19 for over a year, for a variety of situations including international travel. However, supported isolation for returning travellers had, to our knowledge, never been used before within the UK. It was anticipated that the experience could have considerable psychological consequences for the individuals concerned, including potential post-traumatic stress, anger and confusion; consequences that may be affected by a range of stressors including information provision, stigma, and fear of infection [7]. Furthermore, supported isolation represents a unique social context in which relative strangers are placed in close quarters within a novel context and asked to adhere to recommended behaviours for a prolonged period. During emergencies, such social contexts can affect individuals' social identity, which can have consequences for adherence and psychological resilience [8,9,10]. Outside of the emergency response context, the emergence of strong social connections among strangers in close physical proximity has been associated with positive well-being related outcomes [11].

From 15th February 2021 those travelling to the UK from 'red list' countries (countries which have higher prevalence of new COVID-19 variants) [12] have been required to isolate in hotels for 10 days [13]. Countries on the 'red list' are continually reviewed and updated, but as of 9th April 2021 there were 39 countries on the list [14]. Policy around this isolation is focused on identifying the best ways to maximise compliance, with an increasing emphasis on enforcement [15]. Furthermore, with the COVID-19 pandemic ongoing, it is possible that supported isolation will be required in other contexts, such as to assist those with difficulty isolating at home [16] or to reduce household transmission [17]. It is therefore important to understand more about the way in which people experience supported isolation, so that this process can be optimised to increase adherence and mitigate any negative effects on wellbeing. We carried out a rapid mixed-methods study in which we: 1) interviewed individuals who underwent supported isolation at Arrowe Park Hospital and Kents Hill Park conference centre (findings reported here); 2) surveyed those who underwent supported isolation at two time points (immediately after supported isolation and three months after supported isolation) (findings reported elsewhere; Carter et al., in prep). To our knowledge, this is the first research conducted with individuals during and immediately following their supported isolation in this country. With supported isolation now being required for people travelling to the UK from a number of

For peer review only - http://bmjopen.bmj.com/site/about/guidelines.xhtml

countries, the findings presented here will be invaluable in understanding public experiences of supported isolation and informing optimised management in these settings.

Aims

This study had two aims: 1) to understand the experiences and perceptions of those who underwent supported isolation, particularly in relation to factors that were associated with improved compliance and wellbeing; 2) to inform the development of recommendations for the management of similar supported isolation procedures.

Method

Ethical approval

Ethical approval was obtained from the Public Health England Research Ethics Governance Group (approval no. NR0187).

Patient and public involvement

Given the extremely rapid and responsive nature of this research, it was not possible to involve patients or the public in the development of the study and associated materials. However, staff at the supported isolation facilities were involved from the outset in planning the study and facilitating participant recruitment. Additionally, findings from this study will be shared with participants on publication.

Design

This study used semi-structured interviews to capture participants' experiences and perceptions of supported isolation. The decision was taken to carry out semi-structured interviews (alongside surveys, reported elsewhere; Carter et al., in prep) in order to generate a more in-depth understanding of participants' perceptions and experiences during supported isolation than could be obtained using surveys alone. Telephone interviews took place within one-month after the isolation. The study was designed and carried out in-line with consolidated criteria for reporting qualitative research (COREQ) guidelines [18] (see Appendix 1).

Participants

Participants underwent supported isolation in either Arrowe Park (n = 18) or Kents Hill Park (n = 8) in January and February 2020. The day before leaving supported isolation, all those in the supported isolation facilities were provided with an information sheet about the study by a member of staff at the facility. This included an invitation to take part in a survey (findings reported elsewhere; Carter et al., in prep), as well as the opportunity to take part in an interview. Thus, voluntary response sampling was used, whereby all those who underwent supported isolation were given the opportunity to take part in both the survey (reported elsewhere; Carter et al., in prep) and an interview, and the sample consisted of those who chose to opt-in to the study. To opt-in to the interview part of the study, participants were asked to provide an email address on leaving supported isolation to enable the research team to follow up and arrange the interview. At this point, 69 people provided a contact email address, and all were then contacted separately and invited to take part in an interview. Of these, 26 people (38%) consented to take part in an interview; this sample therefore represents 12.3% of the entire population who underwent supported isolation.

Materials

For peer review only - http://bmjopen.bmj.com/site/about/guidelines.xhtml

An interview schedule was developed to capture in-depth information about individuals' experiences and perceptions of supported isolation, including their: overall experience (e.g. "Tell me about your experience of undergoing supported isolation"); willingness to undergo supported isolation (e.g. "Were you willing to undergo supported isolation"); perceptions of the way the supported isolation process was managed (e.g. "In general, how do you feel the supported isolation process was managed?"); perceptions of others' behaviour during supported isolation (e.g. "How did those in supported isolation behave towards each other?"); experiences after leaving supported isolation (e.g. "How has life been for you since leaving supported isolation?"). See Appendix 2 for a copy of the interview schedule.

Procedure

Each interview took place within one month of leaving the supported isolation facility and lasted for approximately an hour. Interviews were carried out by behavioural scientists based at Public Health England or King's College London, all of whom were qualified to at least MSc level and had received training in carrying out interviews. Researchers did not establish a relationship with participants prior to carrying out the interview nor were participants made aware of any personal characteristics of the interviewer, aside from their place of work and the broad aims of the research. Interviews were carried out by both male and female members of the research team. Only the researcher and the participant were present during the interview. Prior to taking part in an interview, participants completed a written consent form. They also provided verbal consent at the start of the interview. Interviews were recorded and subsequently transcribed. After taking part in an interview, participants received a debriefing statement which provided further information about the study, as well as sources of support that participants could access if required.

Analysis

All interviews were completed before beginning data analysis, at which point a framework approach was used to analyse the data [19]. This is a type of thematic analysis that is commonly used within research that has implications for policy and practice [20]. After familiarisation with the data, an initial coding framework was developed based largely on a priori areas of interest in line with the research aims, and specifically included factors that have been shown during previous incidents to be related to compliance and wellbeing.

At this stage, themes were also allowed to emerge from the data. The initial coding framework was intentionally broad, to ensure that areas of interest were not missed, and contained a total of 76 categories, within 22 major themes. The initial framework was discussed with a second researcher, who had also familiarised themselves with the data, and then applied to a small number of transcripts. The initial coding framework was then refined into an analytical framework, in which codes were grouped together into overarching themes. This resulted in 6 key themes, and 7 sub-themes. See Table 1 for a full breakdown of themes and sub-themes.

Application of the analytical framework was carried out by hand by the first author, with each passage in the data being coded into one or more of the identified themes. A spreadsheet was used to generate a matrix into which relevant data (e.g. passages of interest relating to each theme) were organised thematically. This enabled data to be compared and contrasted within and between themes and facilitated more in-depth interpretation. After analysing the 26 transcripts no new themes emerged, thus data saturation had been reached [21].

Results

Demographics

Half of the participants (n = 13) were male and half (n = 13) were female. Participants ranged in age from 22 to 78 (mean = 43.2 years). The majority of participants were British nationals (n = 22), with a small number

For peer review only - http://bmjopen.bmj.com/site/about/guidelines.xhtml

of Chinese nationals (n = 3) and one person who selected 'Other' as their nationality. Similarly, the majority of participants were White British (n = 17), or Chinese (n = 7), with one person being White Irish, and another being Black British. Most participants were educated to degree level or above (n = 17), with a smaller number being educated to higher secondary level (n = 8), and one being educated to primary or lower secondary level. The majority of participants were employed either full time (n = 14) or part time (n = 4). A small number were retired (n = 4), unemployed (n = 2) or self-employed (n = 1), with one participant specifying that they were due to start work following their isolation.

Participants were asked what their reason was for being in Wuhan during the COVID-19 outbreak, and most stated that they were either living there (n = 6), visiting family or friends (n = 8), or on holiday (n = 5). A smaller number were there on a business trip (n = 2), with one participant having been deployed as part of the FCO response. A small number stated that they had not been in Wuhan and were isolating on their return from other affected areas, including Hubei province (n = 2) and the Diamond Princess cruise ship (n = 2). The majority of participants were travelling either with family (n = 11) or on their own (n = 10), with a small number traveling with others they had no relationship with (n = 5). The majority of participants did not share a room (n = 15). Of those that did (n = 11), most shared with family (n = 7) or friends (n = 1), with only a small number sharing with people they didn't know (n = 3).

Table 1: Description of themes and sub-themes

Theme	Sub-theme
Factors affecting compliance	Factors promoting compliance<br>Factors threatening compliance
Risk perceptions around catching COVID-19	Low perceived risk<br>High perceived risk
Management of supported isolation	Operational management<br>Treatment by staff<br>Communication from staff
Communication with those outside	
Relationship with others within supported isolation	
Feelings after leaving supported isolation	

Factors affecting compliance with supported isolation

Factors promoting compliance

For peer review only - http://bmjopen.bmj.com/site/about/guidelines.xhtml

Most participants were willing to undergo supported isolation. They understood why supported isolation was necessary and why they were being asked to undergo it e.g. “I understood the necessity and I was willing to cooperate very much” (KHP2). Most participants felt that the positives of supported isolation outweighed the negatives. Positive aspects were grouped broadly into three themes: a belief that supported isolation protects family and friends as well as UK society e.g. “it was in our best interests and the people we love in the UK and the country in general” (KHP8); a belief that supported isolation would protect themselves, by ensuring they were in a safe place if they developed symptoms and that they would not be blamed in the event of an outbreak in the UK e.g. “in the event that I or any of my fellow travellers developed symptoms we would be in that hospital environment or we would be with doctors who spoke our native language” (AP9); and faith in the effective management of the supported isolation process e.g. “when we actually arrived at Arrowe Park […] the staff there gave such a warm welcome and made everything feel so sort of warm and comfortable” (AP16).

Factors threatening compliance

Where participants expressed concerns these centred around uncertainty about what the process would involve e.g. “You’re thinking well what are the facilities here going to be like? How am I going to cope with that?” (AP24), sometimes attributing this to lack of information being provided e.g. “I was a little bit apprehensive just because I didn’t know […] how it would be structured or organised, and obviously the lack of details” (AP11). Others were concerned that they would be bored, e.g. “[I was concerned that] I would be a bit bored” (AP19) or would be at increased risk of catching COVID-19, e.g. “Our biggest concern would be is anybody sick because of this virus among us?” (KHP2).

A few felt angry or frustrated about the process, because they didn’t think it was necessary e.g. “we did think it was unnecessary because we were already tested negative” (AP24) or believed it was a waste of time and resources e.g. “it was an over the top response that probably cost 2 or 3 million pounds for those two weeks” (KHP4). In the few instances where participants did not want to comply, non-compliance took the form of breaking the rules inside the supported isolation facility (e.g. trying to obtain more alcohol than was allowed), but not trying to leave the supported isolation facility e.g. “Over a short period of time it was let’s try and break the rules just for something to do. Let’s see how far we can go” (KHP4).

Risk perceptions around catching COVID-19

Low perceived risk

Participants’ reported different perceived risks of catching COVID-19 whilst in isolation. Some felt at low risk because they could take protective behaviours e.g. “we were just very careful with washing our hands […] just sensible hygiene precautions really. So that made us feel pretty safe” (AP16). The most commonly reported protective behaviours included staying in their own room e.g. “we just decided not to go out, just to stay in our hotel rooms” (KHP2), observing effective hand hygiene e.g. “I would wash my hands when I went downstairs” (AP11), and wearing a face mask e.g. “we were wearing gloves and masks and keeping no more contact with each other” (KHP3). Other reasons given for low perceptions of risk included that anyone displaying symptoms could be quickly isolated e.g. “I knew that things were being monitored very carefully and things were being done about it” (KHP4), and that everyone in the supported isolation facility underwent regular testing e.g. “after one week we’d all been tested negative, after 10 days we’d all been tested negative, after 14 days we’d all been tested negative” (KHP4).

High perceived risk

However, others were very worried about catching COVID-19 during their stay in supported isolation. Common reasons for this included other people having symptoms e.g. “someone with a high temperature,

For peer review only - http://bmjopen.bmj.com/site/about/guidelines.xhtml

she was really close to me, so I said oh please don't stay too close" (AP10), and the need to sometimes be in close proximity to others e.g. "we were using the same big meeting room for one or two hours before we eventually went to our separate rooms" (KHP2). However, most participants stated that their risk perception reduced over time in the facility, as people continued to test negative, and did not have any symptoms e.g. "towards the end of the isolation, it was getting clearer that nobody in there was probably carrying the virus […] you didn't feel like there was a threat of catching anything from anybody" (AP16). The majority of participants noted that they felt most worried at the start of supported isolation process.

Management of supported isolation

Operational management

Most participants reported that they felt the whole process was well-managed. Reasons for this included that the process was well-organised e.g. "the place all sort of ran like clockwork from my point of view" (AP16), and that staff and management were willing to adapt procedures following negative feedback about the process e.g. "the food initially it was only microwave meals but that evolved in the second week […] everybody was sort of learning as we went along" (KHP5).

Where participants did express concerns these often centred on provision of food, for example not receiving meals e.g. "they forgot to give me breakfast and lunch three times" (KHP1), food being served uncovered e.g. "I think most of us had the salad or the bread which was not covered" (KHP2), and food not being warm enough e.g. "the food turned up lukewarm in cardboard boxes" (AP22). Relatedly, several participants felt that the cultural background of those undergoing supported isolation had not been properly considered. For example, many travellers were Chinese nationals and fresh food is very important to people in China e.g. "when they chose a facility that didn't have fresh food on site they didn't understand the Chinese way of life" (KHP8); the ready meals and pre-prepared foods provided in the first few days of supported isolation were therefore inappropriate.

Another area of management that participants suggested could be improved was around internal communication within and between organisations e.g. "With the change of shifts, they didn't update people […] there was no passing on of communication, there was no register of requests from room numbers (AP18). A final consideration raised in relation to operational management of supported isolation was that several participants would have liked more access to outside space and exercise facilities e.g. "outdoor space improvements may have been helpful […] I think we are all finding value in still being able to get outside a little bit (AP12). For the most part, participants who provided negative feedback about the operational management of the supported isolation process felt that changes were made to address their concerns, and that the management of the supported isolation process improved as time went on e.g. "they are improving their responding and they are learning from their mistakes as well they were really good I was really impressed" (KHP6).

Treatment by supported isolation staff and authorities

Overall, participants were extremely positive in their feedback about the way in which staff treated them. The staff were friendly and helpful e.g. "we were treated with compassion […] and so we were immediately put at ease" (AP24), went out of their way to keep people happy e.g. "the staff went above and beyond in trying to help us" (AP11), and provided people with anything that they asked for e.g. "staff were very helpful, whatever we asked they tried to answer, and whatever we needed they tried to procure" (AP13). A few participants mentioned that staff did not try to avoid them or treat them as if they were ill e.g. "we don't feel that really we were isolated or we were frightening […] as somebody who might carry a virus" (KHP2). A small number of participants specifically noted that staff achieved a good balance between promoting good

For peer review only - http://bmjopen.bmj.com/site/about/guidelines.xhtml

public health, without making the process too restrictive e.g. “I think that’s a balance that had to be struck between health risk and […] how we felt that we were being treated, how restricted we felt” (AP11).

Communication from staff during supported isolation

Participants were also overwhelmingly positive about the way in which members of staff communicated with them. Almost all participants talked about the daily newsletter that they received from staff and felt that this was an effective way of providing information about protective actions, timings of any activities, and testing e.g. “I think they were really good…we would get two or three letters a day actually sometimes about what was changing and why” (AP13). Similarly, participants noted that staff were proactive in their communications, calling regularly to check on each individual e.g. “in the mornings when a nurse would come around […] if there were any sorts of developments to tell us about then they would” (AP15), and scheduling regular update meetings. Participants also felt that staff answered all their questions (or tried to) and were open and transparent in providing information e.g. “I think they would have answered anything that we needed to know” (AP22).

Some expressed dissatisfaction at the somewhat old-fashioned methods of communication e.g. “their way of disseminating information was posting things under the door, which […] seems a little old-fashioned […] maybe if they had done a group chat or done a group email […] I think that may have been a good way of communicating” (AP16), and information not being provided in multiple languages e.g. “the Mum […] had to ask for a lot of help because of her difficulties with English, she was a Chinese national” (AP11). A small number of participants also felt that staff had been unable to answer some questions e.g. “the only information [that staff couldn’t give me] was sort of about leaving actually, and what was going to happen […] that information was only very near the end” (AP15).

Communication with those outside of supported isolation facilities

Most participants found it easy to communicate with those outside supported isolation and did so regularly. Several participants expressed how important this was in helping them to get through the supported isolation process e.g. “we spent half the day usually emailing and skyping and WhatsApping everybody […] it was actually good having that routine” (AP24). Additionally, some were able to carry on working during supported isolation, and this helped them to pass the time. A few participants also highlighted the benefit of local community groups who posted pictures of uplifting things e.g. “it’s nice when you are in that situation […] to see stuff that wasn’t about the virus, and wasn’t doom and gloom” (AP25).

On the other hand, some participants did note difficulties in communicating with those outside of supported isolation, and these typically related to having limited access to internet or poor phone signal e.g. “the phone signal where we were was terrible” (AP22).

Relationship with others within supported isolation

Where people felt a connection with others this was often due to a sense of camaraderie e.g. “I think there was a bit of camaraderie […] everyone was in the same situation really” (AP16) or shared experience e.g. “we were all in the same boat […] it was just, we were all in it together really” (AP22). Some participants described how people supported and encouraged each other during the supported isolation process e.g. “we look after each other, we tried to be helpful with each other as well” (KHP3), stating that this helped people to get through the experience e.g. “we encouraged each other and things like that sometimes. It was good to help many to spend the long and sometimes worrying days” (KHP2). This connection was facilitated by the

For peer review only - http://bmjopen.bmj.com/site/about/guidelines.xhtml

formation of chat groups e.g. “we would message on Facebook and WhatsApp and all that stuff” (AP23), and some level of freedom to socialise with others e.g. “we had a little common room within our side of the conference centre […] so we did movie nights and quizzes and things like that” (KHP5).

Where people did not feel a connection with others this was because they either didn’t get the opportunity to interact much with others, or actively avoided it (due to fears about catching COVID-19) e.g. “they all got together and things like that and the invitation was open but at the same time I didn’t really want to be in the same room with lots of people” (AP13).

Most participants felt that they could trust others to behave appropriately and instances of uncooperative behaviour were rare or non-existent e.g. “people were very very well-behaved […] people are grateful that was a common feeling” (KHP6). A handful of participants noted isolated instances of uncooperative behaviour e.g. “there’s only one argument that we ever heard in the whole two weeks and it was somebody saying that they’ve been tested negative three times can they go home early […] but apart from that the whole two weeks was like with no issue at all (KHP8), but almost all said that the majority of people were friendly and cooperative “Almost all […] were quite cooperative […] I think they were quite friendly to each other” (KHP2).

Feelings after leaving supported isolation

Many participants felt happy and relieved to leave supported isolation and get back to normal e.g. “I’ve never been so happy to see my own bed […] and my own house” (AP16). Most people felt that others had treated them normally on leaving supported isolation, and that they hadn’t experienced negative reactions from others e.g. “nobody has reacted any different to me” (AP17).

However, several participants stated that they struggled after leaving supported isolation. Some felt anxious or overwhelmed, with reasons including not being used to going outside e.g. “I actually had a panic attack when I got in the taxi I found everything very overwhelming […] I hadn’t really mentally prepared myself for going outside” (AP23), or being concerned about mixing with large numbers of people again e.g. “First time we went to the supermarket […] just seeing people who were not in masks and protective clothing took some getting used to […] all the crowds of people in the supermarket when we’d just been used to us two was quite uncomfortable” (AP24). Others simply stated that they had generally struggled on leaving e.g. “the last night we were there, there was no sense of jubilation […] it was just very quiet, very subdued. [Leaving] affected me quite badly really […] I was absolutely lost” (KHP4), or that they had experienced negative reactions from others e.g. “the driver who came to pick us up said ‘I will have to call head office to get the car disinfected after I drop you off’ – that response I think will stay with me for a long time” (AP13).

The majority of participants did not receive follow up information, though a few did receive information about sources of further support. While some stated that they would not have expected to receive any additional information, others felt that this would have been helpful e.g. “I understand there is a lot happening right now…but I don’t think there was enough support for us leaving” (AP23).

Discussion

This paper is the first in-depth analysis of the experiences of those who underwent supported isolation in the UK during the first wave of the COVID-19 pandemic. The findings therefore provide a unique insight into the way in which members of the public perceive supported isolation in the UK, and the factors that affect compliance and wellbeing in such settings. Given that supported isolation is once again required in the management of COVID-19 in the UK [12,13,15], our findings should help facilitate optimised management of supported isolation procedures.

For peer review only - http://bmjopen.bmj.com/site/about/guidelines.xhtml

Despite some initial concerns, including confusion about what the process would involve and fears of infection, all willingly complied with the voluntary supported isolation process. People understood why it was necessary and believed that doing so would protect themselves, their friends and family, and others in the UK; motivation for adherence was largely altruistic. Participants were overwhelmingly positive about their treatment by staff, communication from staff, and overall management of the supported isolation process. This was fundamental to participants' willingness to comply with the restrictions of their liberty. Our findings are in line with systematic reviews carried out at the start of the pandemic [7,22], as well as research into the management of other types of emergencies [8,23]. Crucially, participants believed their treatment by staff was legitimate, and they therefore chose to comply with supported isolation procedures; it is likely that compliance would have been much lower had staff attempted to enforce compliance [23].

There were mixed views as to whether people in isolation experienced a connection with each other. However, almost all reported that others were helpful and friendly. Additionally, a number of people developed a shared identity with others; for example, they talked about everyone being in it together or going through the same experience. Those that did develop a shared identity often reported that this helped them to get through the process. This is as would be expected based on previous research which suggests that when people experience a sense of shared identity with others, this promotes adherence to protective measures, resilience and well-being [8,9,11]. While a sense of shared social identity arose spontaneously in some instances, participants emphasised that being able to communicate with others (for example, via chat groups) enhanced the social support that they experienced. Promoting virtual interaction between those undergoing supported isolation may be beneficial for strengthening shared identity, facilitating provision of social support, and promoting resilience and well-being. Further research could examine how best to employ virtual methods (e.g. WhatsApp groups, social media) to foster shared social identity and social support amongst those undergoing supported isolation, and the impact that this might have on experiences and behaviours during supported isolation. Participants also highlighted how important it was that they were able to easily keep in touch with friends and family during the supported isolation process.

While most participants reported either positive or neutral experiences during supported isolation, it was interesting to note that some reported negative experiences on leaving supported isolation. Findings suggest that it may be beneficial to prepare those undergoing supported isolation for possible psychosocial reactions they may experience upon leaving supported isolation (e.g. feeling anxious or overwhelmed), assist them with logistical aspects associated with leaving supported isolation (e.g. organising travel home, contacting loved ones), and signpost them to sources of support. These would address many of the negative experiences upon leaving supported isolation. However, some participants stated that they had struggled on leaving supported isolation but were not able to explain why that was the case. Further research should be carried out to better understand why some individuals may struggle on leaving supported isolation and improve support to these individuals.

The supported isolation carried out in January – February 2020 was designed to support those who were returning to the UK, and every effort was made to ensure that their experience was as positive as possible; as participants noted, staff could not do enough for them. Isolation in hotels is likely to be very different, with limited support from staff and an emphasis increasingly on enforcement rather than encouragement [15]. The reasons why people are travelling in the middle of a pandemic will also be different. The UK may find itself placing people into isolation who are more likely to experience distress such as those who are arriving to attend a funeral, are travelling due to a family crisis, or who do not speak English. We must also not forget that, unlike travellers placed into facilities at Arrowe Park or Kents Hill, returning travellers will now be asked to pay £1,500 each towards their isolation.

It is therefore critical that those responsible for implementing policies on isolation requirements take into account the recommendations presented here; failure to do so is likely to reduce adherence to isolation and

For peer review only - http://bmjopen.bmj.com/site/about/guidelines.xhtml

risks serious long-term impact on those involved. Further research should explore travellers' experiences of undergoing supported isolation within one of the designated hotels. Due to the key differences (outlined above) between these hotels and the supported isolation reported in this paper, this should be compared with the experiences of those who underwent supported isolation at Arrowe Park of Kents Hill Park, and further our understanding of factors affecting compliance and wellbeing in supported isolation settings.

Limitations

We have no information on those who did not participate, and it is possible that they differed on key variables. Of those that did, we reached thematic saturation within the sample. A second limitation is that only those who had a good understanding of English were interviewed. It is possible that the experience differed for those who were less able to understand English; indeed, this was alluded to in some comments made by participants. A final limitation is that this study was jointly run by King's College London and Public Health England, and Public Health England also assisted with the management of the supported isolation process. The team carrying out this research were not associated with the management of the supported isolation process, although did provide advice to the teams involved. It is therefore possible that participants were aware that PHE played a role in managing the supported isolation process.

Conclusion and Recommendations

Our findings, viewed in the context of the wider relevant published literature, generate several key recommendations that are particularly relevant given the upcoming requirement for travellers to isolate in hotels. Specific recommendations are: 1) prior to supported isolation, authorities should communicate with those affected about why isolation is necessary, how it will help to protect others, and what the process will involve. Given that compliance is often motivated by altruism, emphasising how isolation will protect others is crucial. Such communication will also reduce concerns related to uncertainty about the isolation process; 2) authorities should communicate effectively with those undergoing isolation, throughout the process. Communication should be open and honest, and information should include protective actions people should take, why taking such actions is effective, and how taking such actions protects oneself and others; 3) enforcement of isolation should be avoided wherever possible. Given the large numbers of people who may be required to isolate at one time it will not be possible to enforce adherence; attempting to do so is likely to be perceived as illegitimate, thereby reducing adherence and risking serious long term consequences for those involved; 4) it is likely to be helpful to facilitate and encourage development of shared identity among those undergoing supported isolation, via the formation of chat groups or other means of communication, that include staff managing the facilities. This type of shared social identity should encourage both adherence to supported isolation measures, and improved resilience during the supported isolation process; 5) it is important to ensure that all essential supplies (such as food, exercise facilities, ability to communicate with those outside isolation) are provided and are suitable for the needs of the traveller; 6) authorities should provide relevant information prior to leaving supported isolation to help people to prepare to return to their normal lives. Relevant information should cover the emotions that people might experience, and sources of further support that people can access if required. It may also be beneficial to include in this information any ongoing expectations around adherence to protective behaviours.

For peer review only - http://bmjopen.bmj.com/site/about/guidelines.xhtml

Competing interest statement

All authors have completed the ICMJE uniform disclosure form and declare: HC, DW, IO, CR, and RA are current employees of Public Health England; GJR participates in the UK's Scientific Advisory Group for Emergencies and its subgroups.

Transparency declaration

The lead author affirms that this manuscript is an honest, accurate, and transparent account of the study being reported; that no important aspects of the study have been omitted; and that any discrepancies from the study as originally planned (and, if relevant, registered) have been explained.

Contributor statement

RA, HC, DW, NG, GJR, IO and SW conceived the study. HC, CR, DW and RA collected the data. HC and LG carried out data familiarisation and developed the coding framework. HC carried out the analysis and wrote the first draft of the manuscript. All authors contributed to the design and implementation of the study, and to the writing of the manuscript.

Data availability statement

Data are available on request from holly.carter@phe.gov.uk.

Funding statement

This study was funded by the National Institute for Health Research Health Protection Research Unit (NIHR HPRU) in Emergency Preparedness and Response (grant number 200890), a partnership between Public Health England, King's College London and the University of East Anglia. DW, IO, CR and RA are supported by the NIHR HPRU in Behavioural Science and Evaluation, a partnership between Public Health England and the University of Bristol. CR is also supported by the NIHR HPRU in Emerging and Zoonotic Infections and NIHR HPRU in Gastrointestinal Infections. The views expressed are those of the authors and not necessarily those of the NIHR, Public Health England or the Department of Health and Social Care.

For peer review only

References

1. BBC News. *Coronavirus: Britons evacuated from Wuhan arrive at quarantine facility*. BBC News [newspaper on the Internet] 2020 Jan 31 [cited 2021 Feb 2]. Available from: https://www.bbc.co.uk/news/uk-51318691.

2. BBC News. *Coronavirus: Wuhan Britons end quarantine as cruise passengers isolate*. BBC News [newspaper on the Internet] 2020 Feb 23 [cited 2021 Feb 2]. Available from: https://www.bbc.co.uk/news/uk-51602986.

3. ITV News. *Britons in quarantine after Wuhan evacuation as death toll rises*. ITV News [news website] 2020 Jan 31 [cited 2021 Apr 26]. Available from: https://www.itv.com/news/2020-01-31/evacuated-britons-arrive-at-wirral-hospital-to-begin-fortnight-in-quarantine

4. Li X, Liu M, Zhou R, Zhang Y, Wu C, Xu, L. Centralized medical quarantine for imported COVID-19 in Shanghai, China. *J Travel Med* 2020;27(5):109.

5. Pollack T, Thwaites G, Rabaa M, et al. *Emerging COVID-19 success story: Vietnam's commitment to containment*. Our World in Data 2021 Mar 5 [cited 2021 Apr 26]. Available from: https://ourworldindata.org/covid-exemplar-vietnam

6. Chia ML, Chau DHH, Lim KS, Liu CWY, Tan HK, Tan YR. Managing COVID-19 in a novel, rapidly deployable community isolation quarantine facility. *Annals of Internal Medicine* 2021.

7. Brooks SK, Webster R, Smith LE, et al. The psychological impact of quarantine and how to reduce it: Rapid review of the evidence. *The Lancet* 2020;395(10227):912–920.

8. Carter H, Drury J, Rubin GJ, Williams R, Amlôt R. Applying crowd psychology to develop recommendations for the management of mass decontamination. *Health Security* 2015;13(1):45–53.

9. Drury J, Carter H, Cocking C, Ntontis E, Tekin Guven S, Amlôt R. Facilitating collective psychosocial resilience in the public in emergencies: twelve recommendations based on the social identity approach. *Frontiers in Public Health* 2019;7(141):Doi: 10.3389/fpubh.2019.00141.

10. Drury J, Carter H, Ntontis E, Tekin Guven S. Public behaviour in response to the COVID-19 pandemic: understanding the role of group processes. *BJPsych Open* 2020;1:1–6.

11. Howell JL, Koudenberg N, Loschelder DD, et al. Happy but unhealthy: The relationship between social ties and health in an emerging network. *European Journal of Social Psychology* 2014;44(6):612–621.

12. Department of Health and Social Care. *Booking and staying in a quarantine facility when you arrive in England*. Department of Health and Social Care 2021 Apr 23 [cited 2021 Apr 26]. Available from: https://www.gov.uk/guidance/booking-and-staying-in-a-quarantine-hotel-when-you-arrive-in-england#who-has-to-go-into-managed-quarantine-in-a-hotel

13. BBC News. *Covid: Quarantine hotel plans set to be announced*. BBC News [newspaper on the Internet] 2020 Jan 26 [cited 2021 Feb 2]. Available from: https://www.bbc.co.uk/news/uk-politics-55805575.

For peer review only - http://bmjopen.bmj.com/site/about/guidelines.xhtml

14. Department for Transport. *Coronavirus (COVID-19): red list travel ban countries*. Department for Transport 2021 Apr 23 [cited 2021 Apr 26]. Available from: https://www.gov.uk/guidance/transport-measures-to-protect-the-uk-from-variant-strains-of-covid-19

15. BBC News. *Covid-19: Travellers face £1,750 cost for England quarantine hotels*. BBC News [newspaper on the Internet] 2021 Feb 9 [cited 2021 Feb 9]. Available from: https://www.bbc.co.uk/news/uk-55995645 9th February 2021.

16. Gurdasani D, Bear L, Bogaert D, et al. The UK needs a sustainable strategy for COVID-19. *The Lancet* 2020;396:1800–1801.

17. Denford S, Morton K, Horwood J, de Garang R, Yardley L. Preventing within household transmission of COVID-19: Is the provision of accommodation feasible and acceptable? *medRxiv* 2020.

18. Tong A, Sainsbury P, Craig J. Consolidated criteria for reporting qualitative research (COREQ): a 32-item checklist for interviews and focus groups. *International Journal for Quality in Health Care* 2007;19(6):349-357.

19. Ritchie J, Spencer L. Qualitative data analysis for applied policy research. In: Bryman A, Burgess RG, eds. *Analyzing qualitative data*. London, UK: Routledge 1994. 173 – 194.

20. Pope C, Ziebland S, Mays N. Analysing qualitative data. *BMJ* 2000;320:114–116.

21. Glaser BG, Strauss AL. *The discovery of grounded theory: strategies for qualitative theory*. New Brunswick: Aldine Transaction; 1967.

22. Webster RK, Brooks SK, Smith L, Woodland L, Wessely S, Rubin GJ. How to improve adherence with quarantine: Rapid review of the evidence. *Public Health* 2020;182:163–169.

23. Carter H, Drury J, Amlôt R. Social identity and intergroup relationships in the management of crowds during mass emergencies and disasters: recommendations for emergency planners and responders. *Policing: A Journal of Policy and Practice* 2018:1–14.

For peer review only - http://bmjopen.bmj.com/site/about/guidelines.xhtml

COREQ (COnsolidated criteria for REporting Qualitative research) Checklist

A checklist of items that should be included in reports of qualitative research. You must report the page number in your manuscript where you consider each of the items listed in this checklist. If you have not included this information, either revise your manuscript accordingly before submitting or note N/A.

Topic	Item No.	Guide Questions/Description	Reported on Page No.
**Domain 1: Research team and reflexivity**			
Personal characteristics			
Interviewer/facilitator	1	Which author/s conducted the interview or focus group?	
Credentials	2	What were the researcher's credentials? E.g. PhD, MD	
Occupation	3	What was their occupation at the time of the study?	
Gender	4	Was the researcher male or female?	
Experience and training	5	What experience or training did the researcher have?	
Relationship with participants			
Relationship established	6	Was a relationship established prior to study commencement?	
Participant knowledge of the interviewer	7	What did the participants know about the researcher? e.g. personal goals, reasons for doing the research	
Interviewer characteristics	8	What characteristics were reported about the inter viewer/facilitator? e.g. Bias, assumptions, reasons and interests in the research topic	
**Domain 2: Study design**			
Theoretical framework			
Methodological orientation and Theory	9	What methodological orientation was stated to underpin the study? e.g. grounded theory, discourse analysis, ethnography, phenomenology, content analysis	
Participant selection			
Sampling	10	How were participants selected? e.g. purposive, convenience, consecutive, snowball	
Method of approach	11	How were participants approached? e.g. face-to-face, telephone, mail, email	
Sample size	12	How many participants were in the study?	
Non-participation	13	How many people refused to participate or dropped out? Reasons?	
Setting			
Setting of data collection	14	Where was the data collected? e.g. home, clinic, workplace	
Presence of non-participants	15	Was anyone else present besides the participants and researchers?	
Description of sample	16	What are the important characteristics of the sample? e.g. demographic data, date	
Data collection			
Interview guide	17	Were questions, prompts, guides provided by the authors? Was it pilot tested?	
Repeat interviews	18	Were repeat inter views carried out? If yes, how many?	
Audio/visual recording	19	Did the research use audio or visual recording to collect the data?	
Field notes	20	Were field notes made during and/or after the inter view or focus group?	
Duration	21	What was the duration of the inter views or focus group?	
Data saturation	22	Was data saturation discussed?	
Transcripts returned	23	Were transcripts returned to participants for comment and/or	

For peer review only

Topic	Item No.	Guide Questions/Description	Reported on Page No.
		correction?	
**Domain 3: analysis and findings**			
Data analysis			
Number of data coders	24	How many data coders coded the data?	
Description of the coding tree	25	Did authors provide a description of the coding tree?	
Derivation of themes	26	Were themes identified in advance or derived from the data?	
Software	27	What software, if applicable, was used to manage the data?	
Participant checking	28	Did participants provide feedback on the findings?	
Reporting			
Quotations presented	29	Were participant quotations presented to illustrate the themes/findings? Was each quotation identified? e.g. participant number	
Data and findings consistent	30	Was there consistency between the data presented and the findings?	
Clarity of major themes	31	Were major themes clearly presented in the findings?	
Clarity of minor themes	32	Is there a description of diverse cases or discussion of minor themes?	

Developed from: Tong A, Sainsbury P, Craig J. Consolidated criteria for reporting qualitative research (COREQ): a 32-item checklist for interviews and focus groups. *International Journal for Quality in Health Care*. 2007. Volume 19, Number 6: pp. 349 – 357

Once you have completed this checklist, please save a copy and upload it as part of your submission. DO NOT include this checklist as part of the main manuscript document. It must be uploaded as a separate file.

For peer review only

Appendix 2: Topic guide with questions relating to participants' experiences during and after supported isolation

During supported isolation

Where did you undergo supported isolation?

- Prompts:
    - If you underwent supported isolation at Arrowe Park, what date did you leave?

What were your thoughts about undergoing supported isolation?

- Prompts:
    - Did you understand why you were being put into supported isolation?
    - Did you have any concerns about undergoing supported isolation?
    - If so what were your concerns?
    - Did you think there were any benefits to you of undergoing supported isolation?

Tell me about your experience of undergoing supported isolation.

- Prompts:
    - What has it involved?
    - How has it been?

Were you aware of anyone who was in supported isolation with you having symptoms/ having the coronavirus?

- Prompts:
    - If so, what did you think/ how did you react?

In general, how do you feel the supported isolation process was managed at Arrowe Park/ Kents Hill Park?

Did you feel that staff/ authorities treated you fairly whilst you were in supported isolation?

- Prompts:
    - If so, why?
    - If not, why?

How did you feel about staff/ authority ability to successfully manage the supported isolation process?

Did you trust that the supported isolation process was being managed effectively?

Was there anything that could have been done to improve the way the supported isolation process was managed?

For peer review only

How well do you think staff/ authorities communicated with you whilst you were at Arrowe Park/ Kents Hill Park?

Did staff answer any questions you had?

Did you feel that you were provided with information that you needed?

- Prompts:
  - Were you provided with information about the incident?
  - Were you provided with information about what actions you should take?
  - Were you provided with information about why you were being asked to take certain actions?
  - Was there information you would have liked/ needed that you didn't receive? If so, what?

Did you get the opportunity to communicate with anyone outside of the supported isolation facility?

- Prompts:
  - If so, with who?
  - If so, how did you communicate with them (e.g. phone, social media, email etc)?
  - If so, how often?

Were you willing to undergo supported isolation?

- Prompts:
  - If so why?
  - If not why?
  - If not, what would have made it more likely you would comply?

How do you feel towards the other people who were in supported isolation with you?

- Prompts:
  - Did you feel any connection with them (e.g. bond, shared fate etc)
  - Were you concerned about the possibility of being infected by others?

Did you spend much time with them/ interact much with them?

Did your feelings about the other people affected change over the course of your time in supported isolation?

How did those in supported isolation behave towards each other?

Did you trust that other individuals in supported isolation would behave appropriately?

For peer review only

Did you see anyone refuse to undergo supported isolation?

- Prompts:
  - If so, what action did authorities take?
  - How did other people react?

Post-supported isolation

How has life been for you since leaving supported isolation?

Have you been given follow up information?

Overall, what do you think about the way the Government is managing this outbreak?

Do you feel you have experienced any unhelpful responses by others since undergoing supported isolation?

- Prompts:
  - Do you feel that people have reacted differently to you as a result of your having undergone supported isolation?
  - Do you feel that people have avoided you as a result of your having undergone supported isolation?

Is there anything else you'd like to say that hasn't been covered here?

For peer review only
